# Latitudinal Biogeographic Structuring in the Globally Distributed Moss *Ceratodon purpureus*

**DOI:** 10.3389/fpls.2020.502359

**Published:** 2020-08-28

**Authors:** Elisabeth M. Biersma, Peter Convey, Rhys Wyber, Sharon A. Robinson, Mark Dowton, Bart van de Vijver, Katrin Linse, Howard Griffiths, Jennifer A. Jackson

**Affiliations:** ^1^Biodiversity, Evolution and Adaptation Team, British Antarctic Survey, Cambridge, United Kingdom; ^2^Department of Plant Sciences, University of Cambridge, Cambridge, United Kingdom; ^3^Natural History Museum of Denmark, University of Copenhagen, Copenhagen, Denmark; ^4^School of Earth, Atmospheric and Life Sciences, University of Wollongong, NSW, Australia; ^5^School of Chemistry and Molecular Bioscience, University of Wollongong, Wollongong, NSW, Australia; ^6^Research Department, Botanic Garden Meise, Meise, Belgium; ^7^Ecosystem Management (ECOBE), Department of Biology, University of Antwerp, Antwerp, Belgium; ^8^Ecosystems Team, British Antarctic Survey, Cambridge, United Kingdom

**Keywords:** phylogeography, model organism, moss, spore, wind, bryophyte, global, Antarctica

## Abstract

Biogeographic patterns of globally widespread species are expected to reflect regional structure, as well as connectivity caused by occasional long-distance dispersal. We assessed the level and drivers of population structure, connectivity, and timescales of population isolation in one of the most widespread and ruderal plants in the world — the common moss *Ceratodon purpureus*. We applied phylogenetic, population genetic, and molecular dating analyses to a global (n = 147) sampling data set, using three chloroplast loci and one nuclear locus. The plastid data revealed several distinct and geographically structured lineages, with connectivity patterns associated with worldwide, latitudinal “bands.” These imply that connectivity is strongly influenced by global atmospheric circulation patterns, with dispersal and establishment beyond these latitudinal bands less common. Biogeographic patterns were less clear within the nuclear marker, with gene duplication likely hindering the detection of these. Divergence time analyses indicated that the current matrilineal population structure in *C. purpureus* has developed over the past six million years, with lineages diverging during the late Miocene, Pliocene, and Quaternary. Several colonization events in the Antarctic were apparent, as well as one old and distinct Antarctic clade, possibly isolated on the continent since the Pliocene. As *C. purpureus* is considered a model organism, the matrilineal biogeographic structure identified here provides a useful framework for future genetic and developmental studies on bryophytes. Our general findings may also be relevant to understanding global environmental influences on the biogeography of other organisms with microscopic propagules (e.g., spores) dispersed by wind.

## Introduction

Bryophytes tend to show extensive distributions, often spanning multiple continents and/or both hemispheres (Schofield and Crum, 1972). In particular, the often extremely prolific spore production (Longton, 1997) combined with small spore size (generally ~10–20 μm diameter in mosses; Frahm, 2007) give many bryophytes strong potential for long distance dispersal. Mounting evidence from experimental (e.g. Sundberg, 2013) and phylogeographic studies (e.g. McDaniel and Shaw, 2005; Huttunen et al., 2008; Pokorny et al., 2011; Stenøien et al., 2011; Karlin et al., 2012; Piñeiro et al., 2012; Szövényi et al., 2012; Lewis et al., 2014a; Pisa et al., 2014; Kyrkjeeide et al., 2016; Biersma et al., 2017) has demonstrated the occurrence of long-distance dispersal in mosses with disjunct or widespread ranges, and suggests that the majority of bryophyte distribution patterns are underlain by relatively frequent short distance, and less frequent long distance, dispersal events (Heinrichs et al., 2009; Patiño and Vanderpoorten, 2018; Vigalondo et al., 2019).

Some bryophytes show a truly cosmopolitan or global distribution, similar to some microbial groups (Fontaneto, 2011). The capability for long-distance dispersal in such species is well illustrated by their frequent occurrence on geologically young islands (Convey et al., 2000; Vanderpoorten et al., 2007). Additionally, the speed and frequency with which some of these species colonize newly-erected buildings similarly illustrates the tremendous dispersal potential of such species (Forman, 2014). Yet, the extent to which the global distribution of cosmopolitan bryophytes predominantly reflects recent or ongoing long-distance dispersal events (e.g. over thousands of years), or a worldwide spread acquired over much longer timescales (e.g. millions of years), is poorly known.

*Ceratodon purpureus* (Hedw.) Brid., is one of the most widespread and ruderal moss species known, and can be found in an exceptionally wide geographic range from polar to tropical areas (Ochyra et al., 2008). It is commonly found in harsh, ruderal habitats such as concrete surfaces, buildings, roofs, sidewalks, recently burnt soil and barren glacial deposits (Jules and Shaw, 1994; Shaw and Beer, 1999), and has a high tolerance to drought, pollution, and trampling (Clarke and Robinson, 2008; Forman, 2014; Waterman et al., 2017). The species is commonly used as a model organism in genetic, physiological, and developmental studies, in particular for studying the evolution of developmental processes in bryophytes (e.g. McDaniel et al., 2007, and references therein; Szövényi et al., 2014). For this type of developmental research, good baseline knowledge on the evolutionary history and global biogeography of a species is fundamental, for instance, for underpinning interpretation of crossing experiments, trait mapping and marker discovery, and controlling for demographic or population effects.

The global genetic diversity in natural populations of *C. purpureus* was initially investigated by McDaniel and Shaw (2005) using a worldwide data set of n = 34 samples, including the chloroplast spacer *atpB-rbcL* and the nuclear genes adenosine kinase (*adk*) and phytochrome 2 (*phy2*). They found two distinct Northern Hemisphere clades, a Southern Hemisphere clade including some Northern Hemisphere specimens, and several more distantly related distinct lineages from equatorial regions, suggesting that migration between Australasian and Holarctic regions was more common than among equatorial regions. Overall, the study found that, while markers differed in implied population structure, the overall global population structure in *C. purpureus* was sparse, and provided evidence that migration is ongoing.

Here, we aim to gain a better understanding of the biogeography of *C. purpureus* and assess the level of connectivity between its globally widespread populations. Building on the previous biogeographic analysis of McDaniel and Shaw (2005), we considerably increased the global sampling (n = 147 samples; 257 newly obtained sequences), focusing primarily on three chloroplast markers, the *rps4* gene and *trnL-F* and *atpB-rbcL* spacers and, to a lesser degree, one nuclear marker, the Internal Transcribed Spacer (*ITS*). We tested for global latitudinal and longitudinal population structure, providing an overall assessment of biogeographic patterns in the species. Finally, we assessed the relative divergence time of matrilineal divergent populations, and evaluated timescales over which such widespread populations have been isolated, with a particular interest in those located in isolated, remote regions (e.g. Antarctica).

## Materials and Methods

### Sampling and Molecular Methods

Moss samples for molecular analyses were obtained from herbaria (AAS, BR, BM, E, WOLL, and NY) and fresh collections (see text and **Supplementary Table S1** for sample information). We included sequences available on GenBank, and unpublished sequences from the Honours thesis of RW (Wyber, 2013), originally collected for microsatellite analyses (Clarke et al., 2008, 2009). All specimens were confirmed as *C. purpureus* by specialist bryologists. Genomic DNA was extracted using the DNeasy Plant Mini Kit (Qiagen GmbH, Hilden, Germany), using a mortar and pestle and liquid nitrogen, following the manufacturer’s instructions. As *C. purpureus* has very small gametophytes, multiple stems were extracted per sample to ensure sufficient DNA quantity. The *trnL-F* spacer, the *rps4* gene, *atpB-rbcL* spacer, and *ITS* were amplified using the primers trnLF-c and trnLF-f (Taberlet et al., 1991), trnS (Souza-Chies et al., 1997) and rps 5′ (Nadot et al., 1994), atpB1 and rbcL1 (Chiang et al., 1998) and ITS1 and ITS4 (White et al., 1990), respectively. PCR reactions were performed using the Taq PCR Core Kit (Qiagen GmbH, Hilden, Germany) with addition of MgCl_2_ and Bovine Serum Albumin (BSA), using annealing temperatures of 60°C, 55°C, 53°C, and 55°C for *trnL-F*, *rps4*, *atpB-rbcL*, and *ITS*, respectively. PCR products were checked using gel electrophoresis. For *ITS*, many samples revealed messy or double bands, and for a small selection of samples bands were successfully excised and purified using the Wizard SV Gel and PCR clean up kit (Promega, USA), respectively. Forward and reverse sequencing was performed by LGC Genomics (Berlin, Germany) and the University of Wollongong sequencing facility.

### Sequence Analysis

Forward and reverse sequences were manually checked and concatenated using CODONCODE ALIGNER v.5.0.2 (CodonCode Corp., Dedham, MA). Totals of 74, 94, 80, and 56 concatenated sequences of *trnL-F* (433–435 bp), *rps4* (589 bp), *atpB-rbcL* (623–627 bp) and *ITS* (674–816 bp) were generated, respectively. Sequences were deposited in GenBank as listed in **Supplementary Table S1**. Following McDaniel and Shaw (2005), and based on Hedderson et al. (2004), we selected sequences of *Cheilothela chloropus* (Brid.) Lindb. and three *Dicranum* species from GenBank as outgroups for phylogenetic chloroplast DNA (cpDNA) analyses. CpDNA regions were aligned using the Geneious aligner within GENEIOUS 9.0.4 (Biomatters, LTD, Auckland, NZ). *ITS* was aligned using PRANK (Löytynoja and Goldman, 2008), using default settings. Obvious misalignments were adjusted by eye. In the case of partially incomplete data, short sections at the ends of alignments were excluded. The number of variable and parsimony informative sites of all cpDNA regions were calculated using MEGA7 (Kumar et al., 2016). As McDaniel and Shaw (2005) found evidence for a possible selective sweep, we tested for positive selection in the coding gene *rps4* using the Z-test for synonymous *vs.* non-synonymous mutations, applying the Nei-Gojobori method with Jukes-Cantor correction, within MEGA7 (Kumar et al., 2016).

Three alternative alignments of *ITS* were created; (i) a full PRANK alignment, and with removal of ambiguously aligned or hypervariable regions using (ii) NOISY (Dress et al., 2008) and (iii) GBLOCKS (Castresana, 2000), using default settings. NOISY and GBLOCKS treatments resulted in alignments of length 588–678 and 514 bp, respectively. To test for recombination we applied recombination detection methods within the program RDP v4.71 (Martin et al., 2015) to the original *ITS* alignment, using default settings.

### Phylogenetic and Population Genetic Analyses

For phylogenetic analyses the best-fitting models of evolution were investigated by locus, and by codon for *rps4*, with JMODELTEST-2.1.7 (Darriba et al., 2012) using the SPR tree topology search operation and AICc calculations. This provided the models TIM1+G for *atpB-rbcL* and HKY+G for *trnL-F*. The *rps4* marker was partitioned by codon position with model TPM1uf selected for the first two codon positions and JC for the third codon position.

To estimate population and species relationships, phylogenetic analyses were performed using Bayesian and Maximum likelihood (ML) methods. For individual cpDNA data sets indels were coded with simple indel coding (SIC; Simmons and Ochoterena, 2000) in SEQSTATE v1.0. We investigated four different types of data sets, including i) each cpDNA locus separately (including indel information, where present), ii) a concatenated data set including samples for which all of *rps4* and *atpB-rbcL* loci were complete, called the “*rps4* + *atpB-rbcL*” data set, and iii) a concatenated data set including only samples for which all three cpDNA loci had been sequenced, called the “concatenated cpDNA” data set. This data set was analyzed with and without indels. We also analyzed iv) a data set using all samples and cpDNA loci which had been sequenced, including those samples for which only one or two loci were available.

Bayesian analyses were performed using MRBAYES 3.2 (Ronquist et al., 2012). Runs were continued for 1.5×10^6^ generations, sampling every 1.0×10^3^ generations, and discarding the first 25% as burn-in. Convergence was assessed by confirming that split frequencies had an average standard deviation of < 0.01, and by using TRACER v.1.6 (Rambaut et al., 2014) to confirm that all parameters exceeded effective sample sizes (ESS) > 200. ML analyses were performed using RAxML-GUI v1.3.1 (Silvestro and Michalak, 2012; Stamatakis, 2014), using the “bootstrap + consensus” option (1000 iterations), applying models of evolution most similar to best fitting JMODELTEST-2.1.7 results in each case (e.g. GTR with or without the Gamma model of rate heterogeneity), and applying default settings. Maximum clade credibility trees were visualized using FIGTREE v1.4.2 (http://tree.bio.ed.ac.uk/software/figtree/).

To investigate possible species clusters within the “concatenated cpDNA” data set we used the web-based pairwise genetic distance-based Automatic Barcode Gap Discovery approach (ABGD; Puillandre et al., 2012), applying default settings. ABGD groups samples into hypothetical candidate species based on non-overlapping values of intra- and interspecific genetic distances. We investigated Prior “maximum divergence of intraspecific diversity” (P_max_) values over a range of 0.001–0.05.

To examine phylogeographic structure, TCS networks were produced for all loci, and for the “concatenated cpDNA” data set within POPART (Leigh and Bryant, 2015) using default settings. Within *ITS*, haplotype networks were constructed using original, NOISY and GBLOCKS alignments.

### Population Diversity and Demographic Analyses

To evaluate the cpDNA demographic history, we calculated genetic diversity indices, pairwise Kimura-2P distances, demographic and spatial expansion models, Tajima’s D (Tajima, 1989) and Fu’s Fs (Fu, 1997) neutrality tests for each cpDNA locus with 10000 permutations, using ARLEQUIN v3.5.1.2 (Excoffier and Lischer, 2010). Analyses were performed on the complete data set for each individual cpDNA marker, as well as on the total “concatenated cpDNA” data set. The former were analyzed to investigate population processes using the maximum available sample sizes; the latter using the maximum available co-segregating sites. In the “concatenated cpDNA” analyses we also considered clade substructure within *Ceratodon*, and performed demographic analyses on the overall data set (clades I–VII), and the large ABGD-inferred cluster as defined with P_max_ = 0.0017–0.0046 (clades III–VII) and P_max_ = 0.001 (clades IV–VII).

To investigate worldwide latitudinal and longitudinal geographic structure, we divided the “concatenated cpDNA” data set and the GBLOCKS filtered *ITS* data set into three partitions based on geographical areas. The three *a priori* latitudinal partitions were based on the general atmospheric circulation cells (see Farmer and Cook, 2013) composed of i) 30°S–30°N: the area spanning the Hadley Cells between both Horse Latitudes on each side of the equator, forming one region where air is circulating (via the trade winds); ii) > 30°N; and iii) > 30°S: the Ferrel Cells and Polar Cells at the higher latitudes beyond both hemisphere’s Horse Latitudes, the two other main regions where air is circulating (via the Westerlies and Polar easterlies). The *a priori* longitudinal partitions included regions between 30°W–165°W (Americas), 60°E–30°W (Europe/Africa), and 165°W–60°E (Australia/Asia). We conducted hierarchical AMOVA analysis of partitioning of genetic variation within and between regions in ARLEQUIN v3.5.1.2, and calculated F_ST_ and Φ_ST_ differentiation, using 10000 permutations. Here, F_ST_ estimates reflect differences in composition and frequency between regions, Φ_ST_ estimates reflect levels of evolutionary differentiation between different regions.

### Divergence Time Estimation

To estimate the age of splits among populations we investigated divergence times within the “concatenated cpDNA” data set in BEAST v2.6.2 (Bouckaert et al., 2014). In the absence of suitable fossils we used a relaxed log normal clock with nucleotide substitution rate of 5.0 × 10^−4^ and standard deviation of 1.0 × 10^−4^ substitutions/site/my, respectively; a rate previously applied in bryophyte studies, and corresponding to the average absolute substitution rate of cpDNA across a wide range of land plants and algae (see Villarreal and Renner, 2014, and references therein). We included the same outgroups, models of evolution, and partitioning as described above. To investigate the impact of tree prior choice on divergence times in a data set that contains many population-level samples, as well as potentially different species, we explored two types of tree priors: i) a Coalescent Bayesian Skyline tree prior, and ii) a Yule tree prior. All other parameter settings were identical. MCMC chains were run for 1.0 × 10^8^ generations, with parameters sampled every 10^3^ generations. We combined log and tree files of three runs using LOGCOMBINER v2.6.2 with 10% burn-in. TRACER v.1.6 (Rambaut et al., 2014) was used to assess ESS > 200 for all estimated parameters with 10% burn-in. The maximum clade credibility tree was visualized using TREEANNOTATOR v1.8.2 (Drummond and Rambaut, 2007) and FIGTREE v1.4.2 (http://tree.bio.ed.ac.uk/software/figtree/).

## Results

### Molecular Sequence Data

**Figure 1:**
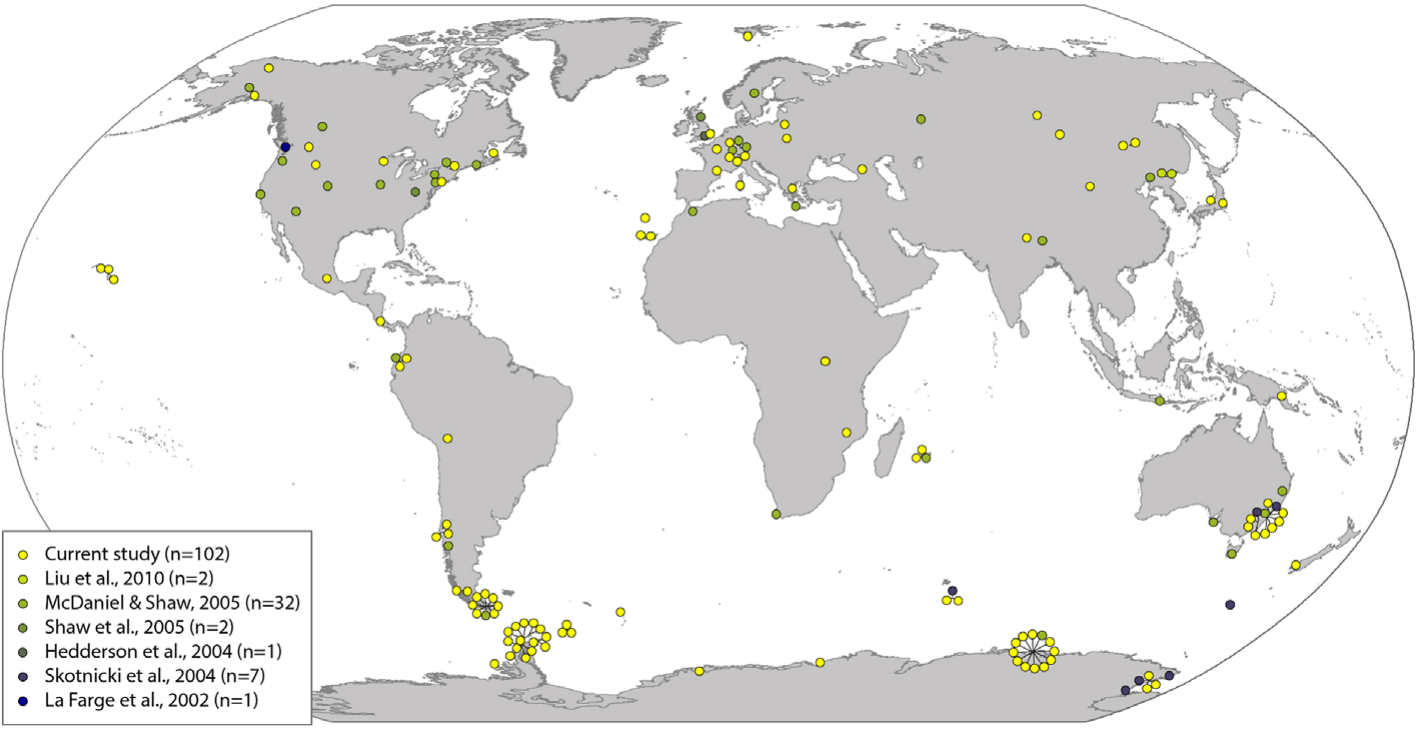
Geographical distribution of *Ceratodon purpureus* samples. Colored dots refer to sequences generated in this study (yellow) and previously published studies (remaining colors; for more information see **Supplementary Table S1**).

The final sample set comprised 147 samples with a wide geographic spread across the globe (distribution shown in **Figure 1**; **Supplementary Table S1**). All cpDNA markers had low nucleotide diversity (π > 0.01) (**Supplementary Table S2**), as would be expected for these markers, which are more commonly used for species- and genus-level rather than population-level studies (Stech and Quandt, 2010). The “concatenated cpDNA” alignment comprised a minimum total combined length of 1,645 bp. Of the cpDNA markers, *atpB-rbcL* was most variable, followed by *rps4* and *trnL-F* (18, 18, and 10 variable sites, and 17, 12, and 7 parsimony informative (PI) sites, respectively). We detected no evidence for positive selection within *rps4* (p > 0.05), but note that low numbers of variable sites limits our ability to draw strong conclusions.

*ITS* had much greater genetic variation than the cpDNA markers, reflected in the numbers of variable (115) and PI sites (61), even after treatment with NOISY or GBLOCKS (83 and 41 variable sites and 60 and 20 PI sites, respectively). Many PCR-amplified samples provided a “clean” single band during electrophoresis (**Table S1**). Other samples yielded double or messy bands, and were not sequenced. Two sets of double bands were successfully excised and sequenced, and revealed multiple copies of *ITS* in the same specimens (samples from Hawaii and Australia, see Discussion for more detail). We did not investigate the occurrence of *ITS* copies further (e.g. through cloning) as *ITS* amplification of other contaminants e.g. fungi, is common in herbarium samples, and this approach would be better pursued through future studies with fresh rather than degraded herbarium material. Recombination tests revealed evidence of recombination signals in five of the seven tests performed (GENECONV, BOOTSCAN, MAXCHI, SISCAN and 3SEQ; Martin et al., 2015).

### Phylogenetic and Population Genetic Analyses

**Figure 2:**
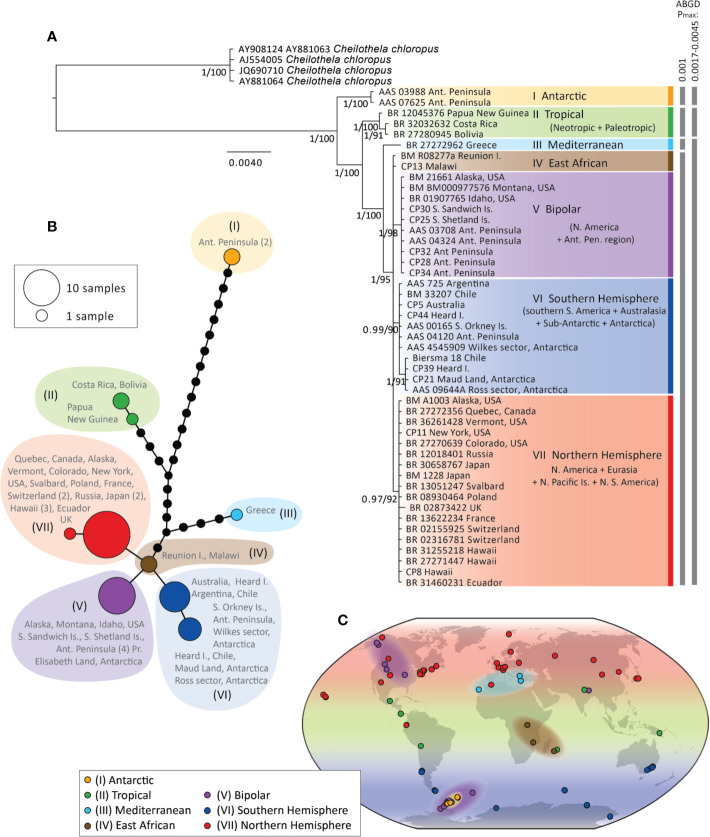
Bayesian phylogeny **(A)** and haplotype network **(B)** for *Ceratodon purpureus* constructed with a concatenated cpDNA data set (*atpB-rbcL*+*rps4*+*trnL-F*). Posterior probabilities and bootstrap support are shown next to branches **(A)**. The scale bar represents the mean number of nucleotide substitutions per site. Biogeographic clade descriptions (I–VII) and ABGD species-clusters with different P_max_-values are shown next to **(A)**. In **(B)** haplotype circle sizes and colors correspond to the number of specimens and clades I–VII, respectively. Branches represent mutations between haplotypes, with mutations shown as 1-step edges. **(C)** represents the sample locations and biogeographic regions of samples in the different clades (I–VII) as interpreted from the concatenated cpDNA data set **(A)**, as well as the placement of samples in phylogenies of single cpDNA markers (e.g. when samples were only represented by one or two single cpDNA marker(s); see also **Supplementary Figures S1A–D**). For example, while the Mediterranean clade (III) includes just one sample (from Greece) in the concatenated cpDNA data set **(A)**, clade III on the map in **(C)** includes one more sample from Greece (AY881059) and one from the Canary Islands (BM 27), based on the well-resolved placement of these samples in clade III in the Bayesian phylogenies of *atpB-rbcL* (**Figure S1A**) for the former, and *rps4* and *trnL-F* (**Figures S1C, D**) for the latter, respectively.

Phylogenetic trees and haplotype networks of the “concatenated cpDNA” data set are presented in **Figures 2A, B**, respectively (for trees and haplotypes of individual loci and the “*rps4* + *atpB-rbcL*” data set see **Supplementary Figures S1** and **S2**). No topological conflicts were found between Bayesian and ML analyses. Phylogenetic relationships were similar for analyses of the “concatenated cDNA” data set with and without indels included (the former not shown).

Phylogenetic analyses of the “concatenated cpDNA” data set revealed seven highly supported clades (posterior probability (PP) > 0.97, bootstrap values > 90) (**Figure 2A**). These included, in order, an Antarctic clade (I), a tropical clade (II), a single-specimen lineage originating from Greece (III), and a polytomy consisting of an East African clade (IV), a bipolar clade (V), a Southern Hemisphere clade (VI) and a Northern Hemisphere clade (VII). All single-locus analyses also resolved the first three clades (I–III) (see **Supplementary Figures S1A–C**), while the latter four clades were resolved only by *atpB-rbcL* and/or *rps4*. The phylogeny with all sequenced cpDNA loci (data set iv) showed very limited resolution by comparison, with most samples falling as a single large polytomy (data not shown).

The geographic ranges spanned by each clade (I–VII) are shown by locus in **Supplementary Figures S1** and **S2**, and visualized in **Figure 2C**. Clade I only included specimens from the maritime Antarctic. Clade II included specimens from equatorial regions in the Palaeotropics and the Neotropics, including Papua New Guinea, Nepal, Réunion Island, Mexico, Costa Rica, and Bolivia. Clade III consisted of specimens from Greece and the Canary Islands. The East African group (IV) was not resolved by individual loci, but appeared ancestral within clades IV–VII (**Figures 2A, B**) and “*rps4* + *atpB-rbcL*” data set (**Supplementary Figure S1D**), and included specimens from Reunion Island, Malawi, and Uganda. The bipolar clade (V) (**Figures 2A–C**) included specimens from western North America, the sub-Antarctic, and Antarctic, as well as one specimen from India (this was only resolved in the *atpB-rbcL* marker; **Figure S1A**). The Southern Hemisphere clade (VI) included specimens from higher latitudes in the Southern Hemisphere (South America, Australia, the sub-Antarctic, and Antarctica). Finally, the Northern Hemisphere clade (VII) included specimens from the Holarctic, Hawaii, and one specimen from Ecuador.

The species delimitation method ABGD revealed three or four significant “barcoding gaps” in the concatenated cpDNA data set at P_max_ = 0.0017–0.0046 (separating I, II, and III–VII) and P_max_ = 0.001 (separating I, II, III, and IV–VII), respectively (see **Figure 2A**). Clades I and II were identified as different species clusters from the rest of the data set in all the P_max_-values analyzed, while clade III was only regarded as a distinct species cluster at the lower P_max_-value.

**Figure 3:**
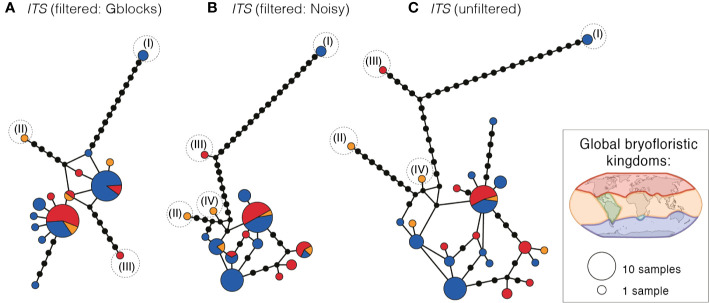
Haplotype networks of *ITS* for *Ceratodon purpureus*, after treatment with **(A)** GBLOCKS, **(B)** NOISY or as **(C)** original data. Haplotype circle sizes and colors correspond to the number of specimens and globally recognized bryofloristic kingdoms (see legend; Schofield, 1992), respectively. Branches represent mutations between haplotypes, with mutations shown as 1-step edges. Numbers (I–IV) indicate the placement of the same samples falling in clades I–IV as resolved in the cpDNA data sets (see **Figure 2**).

Haplotype networks of *ITS* (based on GBLOCKS, NOISY, and original alignments (**Figures 3A–C**); for more detail see **Supplementary Figure S3**) revealed that the same samples representing distinct clades I, II, and III in the cpDNA (see **Figure 2**) were also genetically distinct in *ITS*, with long branch-lengths in all *ITS* networks (**Figures 3A–C**; **Figure S3**). No distinct clustering of the remaining cpDNA-defined clades (V–VII) was apparent in the *ITS* networks. Multiple gene copies within the same sample were placed in widely separated regions in the haplotype networks (indicated with * in **Figure S3**).

### Population Diversity and Demographic Analyses

**Figure 4:**
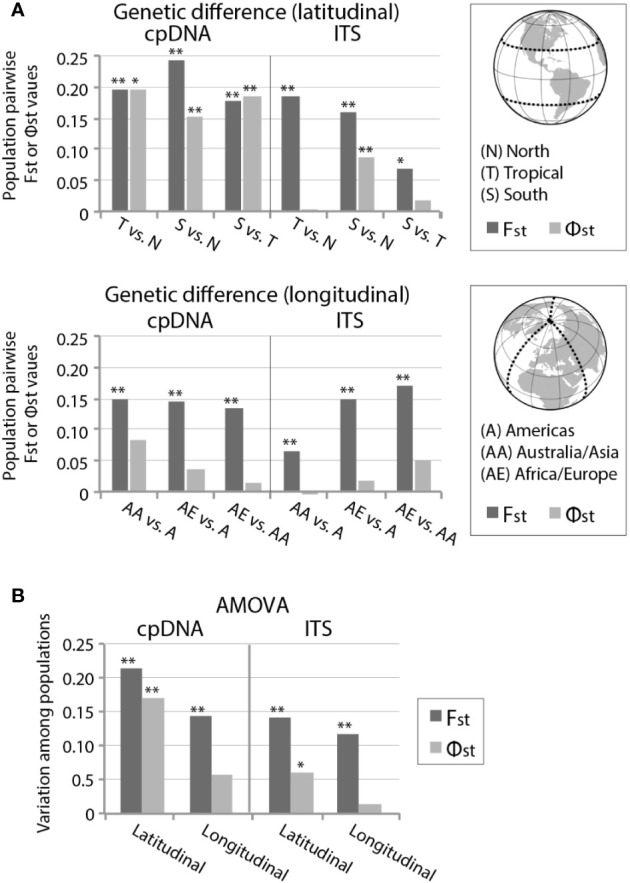
**(A)** Pairwise F_ST_ and Φ_ST_ values and **(B)** analysis of molecular variance (AMOVA) within *Ceratodon purpureus* populations based on latitudinal and longitudinal geographically divided areas, including samples from the “concatenated cpDNA” and GBLOCKS filtered *ITS* data set. For details see **Tables S3–S5**. For geographically divided areas see legend in **(A)**. P-values are represented by * for P < 0.05 and ** for P < 0.01.

F_ST_ and Φ_ST_ calculations of cpDNA revealed significant genetic differentiation between the latitudinal geographic areas (**Figure 4A**; **Supplementary Table S3**). *ITS* also revealed significant genetic differentiation in composition and frequency (F_ST_) between all latitudinally divided regions, but only the northern (> 30°N) and southern region (> 30°S) showed significant genetic differentiation (Φ_ST_) from one another. When divided based on longitudinal geographic regions, both cpDNA and *ITS* showed significant haplotypic differentiation (F_ST_) between regions, but no significant evolutionary differentiation (Φ_ST_) (**Figure 4A**; **Supplementary Table S4**). Latitudinal regionalized partitions of cpDNA revealed Φ_ST_ > F_ST_ in two population comparisons (tropical versus either north or south, **Figure 4A**; **Supplementary Table S4**), suggesting a significant phylogeographic signal. This was not seen in longitudinal regionalized partitions of cpDNA, or in the *ITS* data for either latitudinal or longitudinal partition.

Hierarchical AMOVA analyses of cpDNA estimated that 21% and 17% of haplotypic (F_ST_ = 0.21, *p* < 0.01) and genetic (Φ_ST_ = 0.17, *p* < 0.01) differentiation was found within latitudinal bands, respectively, while longitudinal regionalization showed weaker haplotypic differentiation (F_ST_ = 0.14, *p* < 0.01) and no significant genetic differentiation (Φ_ST_ = 0.06, *p* = 0.07) (**Figure 4B**; **Supplementary Table S5**). *ITS* revealed a less strong but similar pattern, with 14% and 6% of haplotypic (F_ST_ = 0.14, *p* < 0.01) and genetic (Φ_ST_ = 0.06, *p* < 0.05) differentiation found among latitudinal geographic regions, while longitudinal regionalization showed a decreased haplotypic differentiation (F_ST_ = 0.12, *p* < 0.01) and no significant genetic differentiation (Φ_ST_ = 0.01, *p* = 0.22) (**Figure 4B**; **Supplementary Table S5**).

The demographic expansion test on the concatenated cpDNA data set of all clades (I–VII) was significant, rejecting a demographic expansion (**Supplementary Table S2**). The spatial expansion test was non-significant, indicating that the genetic pattern may be consistent with a spatial expansion. Tajima’s D was significantly negative in the full (I–VII) concatenated cpDNA data set, as well as the full data sets of *rps4* and *trnL-F*, suggesting that rare alleles were present in these loci at lower frequencies than expected, indicative of a selective sweep or past population expansion. However, Tajima’s D was not significant when the most divergent lineages (I–II) were removed, suggesting the significant results were mostly influenced by cryptic population structure within *C. purpureus*. Furthermore, Fu’s Fs was not significant for any data set, suggesting that overall cpDNA does not support a rapid past expansion of *C. purpureus* on a global scale.

### Divergence Time Estimation

**Figure 5:**
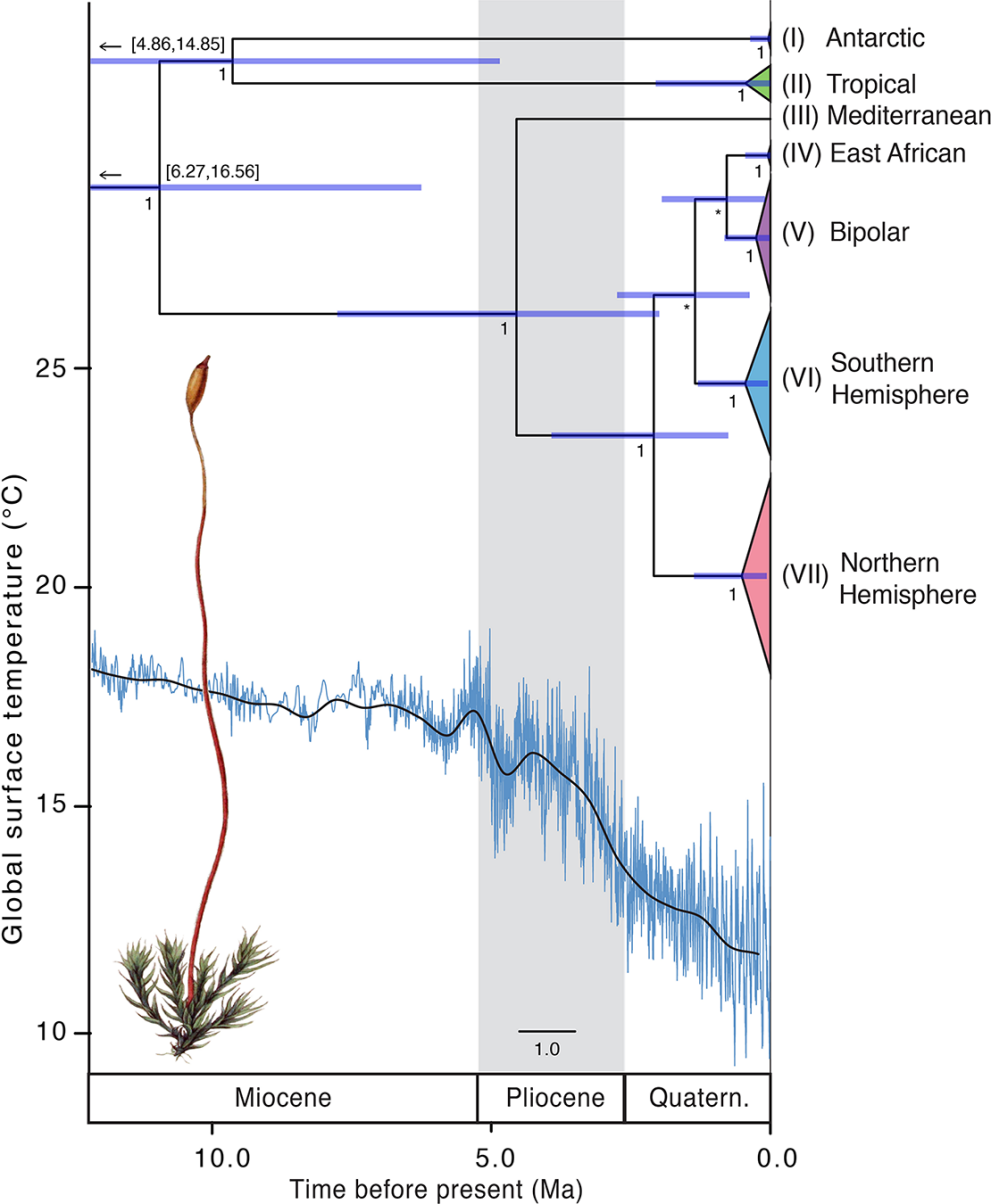
Time-calibrated phylogeny of *Ceratodon purpureus*. The maximum clade credibility tree presents the median divergence time estimates for major lineages (**Figure 2A**) from a concatenated cpDNA data set (*atpB-rbcL*+*rps4*+*trnL-F*) using a coalescent tree prior. Node bars represent 95% height posterior distribution of age estimates. Posterior support (PP) values are shown below nodes, with PP < 0.5 provided as *. Global surface temperature estimates (blue and solid line representing temperature variations and a 500 kyr smoothed resolution, respectively), reproduced from Hansen et al. 2013, are provided below. Due to the old age (62.78 Mya; 95HDP 26.94–91.13 Mya; ) the split of the outgroup *Cheilothela chloropus* and *Ceratodon purpureus* is not shown in this figure. Note that the lower part of the 95HPD range of the two oldest nodes is not shown, but given in numbers above the node bars. Scale bar indicates the number of substitutions per site. Illustration by Christiaan Sepp (Kops et al., 1868; Wikimedia Commons).

The divergence time analysis using a coalescent tree prior indicated that the ancestor of the *C. purpureus* clades originated in the mid- to late Miocene ~11.24 Mya (with 95% highest posterior density intervals (95HPD): 6.27–16.56 Mya; see **Table 1**, **Figure 5**), a time when the Antarctic (I) and tropical (II) clade diverged from the remaining clades (III–VII). The splits between clades III–VII and clades IV–VII (which were, depending on P_max_ value, delimited as one species using ABGD; see **Figure 2**) were dated to be 4.76 (95HPD: 2.00–7.78) Mya and 2.22 (95HPD: 0.76–3.93) Mya, respectively.

**Table 1:** Mean estimated time to most recent common ancestor (T_MRCA_) (95% HDP lower–upper) for clades within the “concatenated cpDNA” data set (see **Figure 2A** ) of *Ceratodon purpureus*, as calculated in BEAST.

Lineage T_MRCA_	Mean ages (my) Coalescent Bayesian Skyline tree prior	Mean ages (my) Yule tree prior
*Cheilothela chloropus* + *Ceratodon purpureus*	62.78 (26.94–91.13)	7.18 (3.90–12.20)
I–VII	11.24 (6.27–16.56)	5.98 (2.68–9.80)
I–II	9.80 (4.86–14.85)	3.99 (0.92–7.11)
III–VII	4.76 (2.00–7.78)	4.66 (1.95–7.85)
IV–VII	2.22 (0.76–3.93)	3.81 (1.54–6.53)
I Antarctic	9.19×10^−2^ (2.21×10^−7^ to 0.36)	0.72 (1.43×10^−6^ to 2.22)
II Tropical	0.68 (2.97×10^−3^ to 2.06)	1.49 (0.07–3.45)
IV East-African	0.11 (2.47×10^−7^ to 0.45)	0.91 (2.33×10^−6^ to 2.56)
V Bipolar	0.33 (1.46×10^−2^ to 0.83)	1.99 (0.49–3.85)
VI Southern Hemisphere	0.55 (4.47×10^−2^ to 1.30)	2.23 (0.61–4.18)
VII Northern Hemisphere	0.61 (6.39×10^−2^ to 1.37)	2.55 (0.84–4.63)

Implementing a Yule tree prior the analysis indicated that the ancestor of the *C. purpureus* clades originated in the late Miocene ~5.98 Mya (95HPD: 2.68–9.80 Mya; see **Table 1**, **Supplementary Figure S4**), and clade III diverged from the other clades ~4.66 Mya (95HPD: 1.95–7.85 Mya). The remaining clades (IV–VII) diverged ~3.81 Mya onwards (95HPD: 1.54–6.53 Mya). Regardless of the tree prior used, the remaining clades (IV–VII) diverged throughout the late Pliocene to mid-Quaternary, also a period of global cooling (Hansen et al., 2013; for a comparison with global surface temperature see **Figure 5**). All nodes had high posterior support (**Figure 5**), except for the node uniting clades IV and VI, and that separating V from IV+VI, relationships which were also not strongly supported in the phylogenetic analyses (**Figure 2A**). The phylogenies of BEAST (**Figures 5** and **S4**) and MrBayes (**Figure 2**) showed a topological inconsistency (with clade I being sister to the remainder of *Ceratodon* clades in the latter, while clades I and II were sister to each other in the former analyses). The analysis in BEAST incorporates a relaxed clock, which has a potential to improve phylogenetic accuracy but on the other hand can be less precise than the time-free approach taken in MrBayes, depending on the true underlying evolutionary pattern (Wertheim et al., 2010). Consequently, we decided to retain both topologies despite the minor biogeographic differences.

## Discussion

### Old Lineages in Distinct Biogeographic Regions

The analysis of the cpDNA loci within *C. purpureus* revealed well-supported, several hundreds of thousands to multi-million-year old lineages derived from distinct global regions (**Figures 2** and **3**). This surprising finding implies that the global distribution of a ruderal, cosmopolitan species such as *C. purpureus* is mainly the result of a worldwide spread achieved by dispersal and establishment over hundreds of thousands to million-year timescales rather than high-frequency long-distance dispersal events, as would be expected for a highly ruderal species. The old age of the genus *Ceratodon* is also in line with previous age estimates implementing fossil ages (Laenen et al., 2014).

The matrilineal lineages were mainly strongly linked with latitude (**Figures 2C** and **4**; **Supplementary Tables S3–S5**). This global latitudinal structuring was particularly evident for cpDNA (significant F_ST_ and Φ_ST_; **Figure 4**), and partly reflected in *ITS* (significant F_ST_, while only one latitudinal comparison showed a significant Φ_ST_). Biogeographic patterns were in line with those found by McDaniel and Shaw (2005), while the increased sampling and additional cpDNA loci considerably expanded the geographic extent and characterization of several clades (particularly newly-recognized clades I and VI).

All cpDNA loci (**Figures 2** and **3**) and the *ITS* marker (**Supplementary Figure S3**) revealed particularly strong differentiation of the first three clades identified (I–III). The ABGD species delimitation method supported a species complex with at least three (I, II, and III–VII) and possibly four (also dividing III from IV–VII) species, with the whole complex referred to as *C. purpureus sensu lato (s.l.)* hereafter.

Several taxonomic studies have previously also noted phenotypic differentiation within geographically separated populations of *C. purpureus* (Burley and Pritchard, 1990; Ochyra et al., 2008, and references therein), observations which regain credence based on the genetic differentiation of cpDNA and *ITS* regions within *C. purpureus s.l.*, and which are of relevance to future developmental studies using the species as a model species.

In their global revision of the *Ceratodon* genus, Burley and Pritchard (1990) identified four species and three subspecies based on a quantitative assessment of morphological characters. Although their work did not gain wide acceptance (Ochyra et al., 2008), the phylogeny and geographic regions of their described species and subspecies show a striking similarity with the genetic patterns obtained in our study. The overlap in geographic regions in the Holarctic occupied by *C. purpureus* ssp. *purpureus* with Northern Hemisphere clade VII suggests an agreement between morphological and genetic evidence. A similar resemblance was found in the occurrence of Holantarctic *C. purpureus* ssp. *convolutes* (Reichardt) J.S. Burley and Southern Hemisphere clade VI. Although ssp. *convolutes* was suggested to be found in southern South America, the sub-Antarctic and Australia (Burley and Pritchard, 1990), we also find many specimens of this clade (VI) in Antarctica. The third described subspecies in Burley and Pritchard (1990), *C. purpureus* ssp. *stenocarpus* (B.S.G.) Dix., found in equatorial regions of the Old and New World, has a distribution consistent with that of tropical clade II.

Other lineages for which distinct overlap was found in both biogeography and phylogenies of work by Burley and Pritchard (1990) included: 1) the bipolar species consisting of the morphologically similar Antarctic *C. antarcticus* Card. and Arctic *C. purpureus* ssp*. arcticus* Kindb., showing an overlap with bipolar clade V; and 2) the distinct European (or Eurasian) *C. conicus* (Hamp.) Lindb., showing a possible overlap with clade III.

The morphological differences in *Ceratodon* between different geographic areas reported by Burley and Pritchard (1990) point towards a phenotypic response to local environmental conditions. It should be noted however, that we did not directly compare our samples with the exact samples used in Burley and Pritchard (1990), nor did we make detailed morphological re-assessments of our specimens, and therefore further morphological assessments are needed to clarify the status of these taxa. However, it is clear that several ancient and geographically distinct lineages within specimens morphologically assigned to *C. purpureus s.l.* exists, a pattern that might be similar in other species that are currently assumed to be globally distributed cosmopolitan species.

### Concordance in Phylogeographic Patterns Between cpDNA and *ITS*

Biogeographic patterns of cpDNA were only partly mirrored by *ITS*. The latter revealed clear differentiation of the earlier diverging lineages (primarily clades I–III; **Figure 3**); however, the remaining clades could not be clearly distinguished, and *ITS* haplotypes could be found across geographically separated areas around the globe (**Figure 3**).

*ITS* and cpDNA revealed concordance in most of the population Φ_ST_ and F_ST_ comparisons according to latitude or longitude. Both genetic compartments revealed significant haplotypic differentiation (F_ST_) in all latitudinal and longitudinal population comparisons. However, while cpDNA showed significant evolutionary differentiation (Φ_ST_) in all latitudinal comparisons (implying a latitudinal biogeographic signal), this was only found in one latitudinal population comparison (> 30°N vs. > 30°S) in *ITS* (**Figure 4A**; **Supplementary Table S4**). Neither cpDNA nor *ITS* showed significant evolutionary differentiation (Φ_ST_) in any of the longitudinal partition comparisons, implying that there is no strong longitudinal biogeographic signal.

This observed difference in geographical patterns and Φ_ST_ values in latitudinal population comparisons between cpDNA vs. nDNA could have several origins. The different nuclear pattern is unlikely to be attributed to migration of male gametes: the latter (unlike pollen in angiosperms) have an extremely limited dispersal capacity in mosses (Pressel and Duckett, 2019). The nuclear pattern is more likely to be caused by paralogy and evolution of pseudogenes. Previous research has shown *C. purpureus* has undergone an ancient nuclear genome duplication (Szövényi et al., 2014), a finding also shown in McDaniel and Shaw (2005) through the different gene copies of *adk* and *phy2*. In addition, *ITS* is known to have paralogous copies in mosses (Vanderpoorten et al., 2006). In our study we also found *ITS* had copies in several samples, and the two samples for which two different copies were sequenced were placed in widely spaced haplotypes in the haplotype network (**Figure S3**). In addition to multiple paralogous copies, the signal of the nDNA is likely influenced by the use of multiple moss shoots in the DNA extraction, and other factors such as recombination (shown in *adk* and *phy2* in McDaniel and Shaw, 2005, and *ITS* in this study), genetic interactions among nuclear loci (McDaniel et al., 2007; McDaniel et al., 2008), and possibly by processes such as hybridization among populations, selection or incomplete lineage sorting (i.e., cpDNA is haploid and therefore is subject to more rapid genetic drift than nuclear DNA, while the nuclear DNA has undergone genome duplication, shows high diversity and extensive recombination).

Previously, McDaniel and Shaw (2005) suggested the cpDNA to have undergone a selective sweep. Our results are consistent with this possibility, with current levels of diversity suggesting a selective sweep prior to the mid-Miocene, with subsequent cpDNA population structure likely reflecting patterns of wind dispersal since this time. In contrast the nuclear genome may reflect much older patterns of dispersal and connectivity over the Tertiary period.

Although our genealogical history of *ITS* is not complete (due to the difficulty of obtaining paralogous copies with degraded herbarium specimens), we highlight the importance of combining nuclear and plastid markers to unravel evolutionary histories, as both are part of a species’ biology. We await future studies on the nuclear genome of *C. purpureus* (using fresh rather than herbarium samples) to advance knowledge on nDNA biogeographic patterns, as well as the extent, causes and consequences of recombination and genome doubling on the evolution in *C. purpureus* and other non-vascular plants.

### Drivers of Dispersal and Establishment

**Figure 6:**
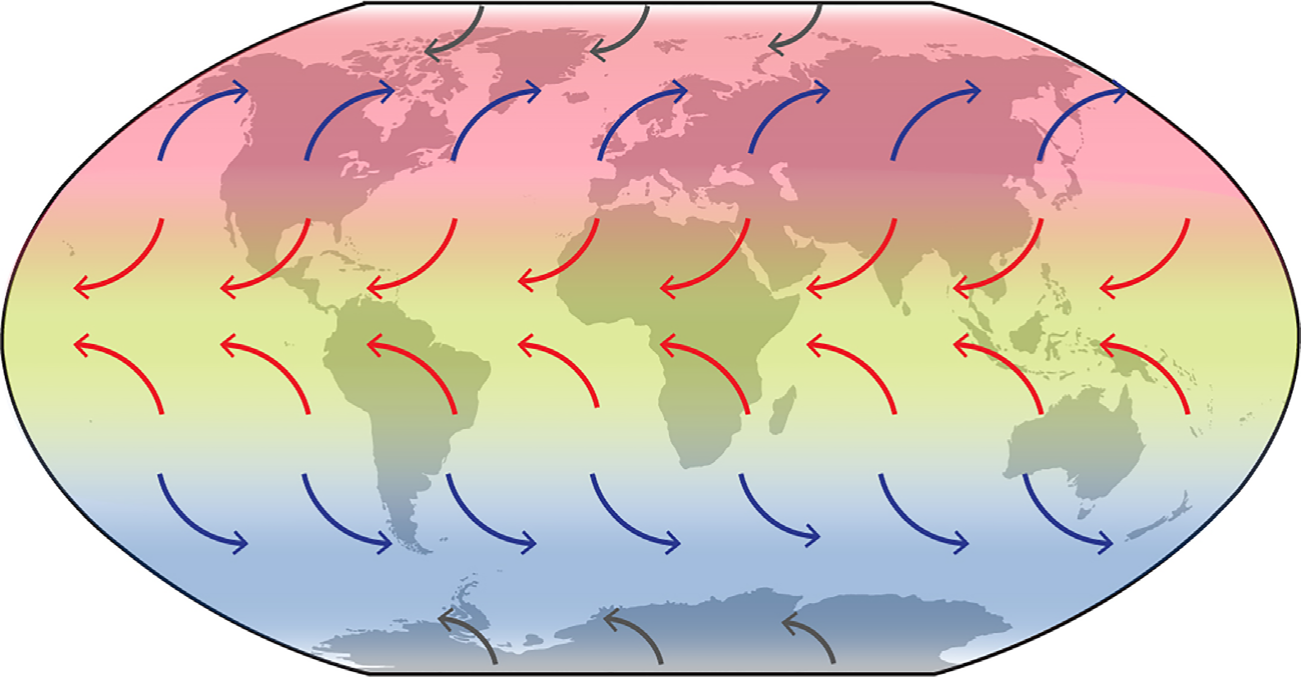
Global wind patterns overlaying the distributions of the Southern Hemisphere (VI), Northern Hemisphere (VII), and tropical (II) clades (see **Figure 2A**). Red arrows: Trade Winds; blue arrows: Westerlies; grey arrows: Polar Easterlies.

We found clear biogeographic structuring dividing the main global populations of *C. purpureus s.l.* into physically distinct “latitudinal” geographic areas (see **Figures 2C** and **4**; **Supplementary Tables S3–S5**). This structuring is plausibly linked with atmospheric circulation patterns (see **Figure 6** for general global wind patterns overlaying the distributions of the Southern Hemisphere, Northern Hemisphere, tropical clades). Generally, global air masses, and thus particles that are carried within them, are retained within particular latitudinal bands or geographic regions. At higher latitudes in both hemispheres, the prevailing westerly winds (blue arrows; **Figure 6**) generally isolate higher latitudes from equatorial regions. These could therefore be an isolating force for the Southern Hemisphere (VI) and Northern Hemisphere (VII) clades. Similarly, the easterly trade winds (red arrows; **Figure 6**) move mostly towards the equator, thus retaining entrained particles within the equatorial band. These could be an isolating force for clade II, found in tropical regions around the globe.

Similar isolating latitudinal distributions *via* wind currents have also been reported in aerobiology modeling studies (Muñoz et al., 2004; Wilkinson et al., 2012). Such atmospheric circulation patterns not only restrict the movement of particles within a given hemisphere or latitudinal band, but could, by doing so, be a driving isolating force triggering adaptation to particular climatic zones. Such genetic isolation in *C. purpureus* has been shown by McDaniel et al. (2007, 2008), who showed that considerable reproductive isolation exists between a tropical and temperate population (from Ecuador and the northern United States, respectively), including genetic incompatibilities within hybrids. If historic distribution of spore-dispersed organisms such as *Ceratodon* has been structured by atmospheric circulation, then climate change and ozone depletion, which are changing the position and strength of such wind belts (e.g. Polvani et al., 2011; Thompson et al., 2011; Turner et al., 2014; Robinson and Erickson, 2015; Robinson et al., 2020), will likely influence dispersal in the future. Increased ice melt is also predicted to increasingly open up new areas for colonization especially in polar and montane regions (e.g. Lee et al., 2017; Robinson et al., 2020).

Dispersal between hemispheres is generally limited due to atmospheric circulation patterns causing the Intertropical Convergence Zone across the equator. The distinct bipolar group (clade V; **Figure 2**) is nevertheless likely a product of at least one long-distance dispersal and establishment event across the equator. Bipolar disjunctions are a distribution pattern characteristic of many bryophytes (Ochyra et al., 2008), however recent molecular studies (e.g. Lewis et al., 2014a, 2017; Biersma et al., 2018b; Shaw et al., 2019) suggest that trans-equatorial movements are infrequent events that happen very occasionally over hundreds of thousands to multi-million year timescales. Such trans-equatorial dispersal could have been facilitated by sporadic air movements or zoochory (adventitious attachment to other organisms, e.g. birds). As nearly all clade V specimens belonged to the New World and the Antarctic region directly south of this (excepting a single specimen from India resolved to this clade in the *atpB-rbcL* marker; **Figure S1A**), migratory birds that link parts of these northern and southern regions in their annual migration are plausible vectors (cf. Lewis et al., 2014b; also a likely vector for bipolar vascular plant species, Villaverde et al., 2017). Whether the placement of the Indian sample in this bipolar clade is the result of a different long-distance dispersal event, a human induced introduction, or perhaps a matter of convergent evolution in the *atpB-rbcL* marker, requires further regional sampling and sequencing to investigate.

As *C. purpureus s.l.* is a ruderal species, characteristically found in a wide range of dry and disturbed habitats, an increase in environmental (e.g. glacial, fire-influenced and, more recently, anthropogenic) disturbances could have aided its spread across the globe. The main distribution of *C. purpureus s.l.*, occupying the vast areas of the temperate regions (particularly clades V-VII), became established throughout the late Pliocene and Quaternary. This was a period of high disturbance globally, including global cooling and repeated glacial periods (Hansen et al., 2013; see **Figure 5**). It is likely that repeated glacial disturbance provided favorable conditions for the spread and population expansion of *C. purpureus s.l.* in high latitude areas of both hemispheres. Additionally, the rise of modern flammable grass-, shrub- and woodlands (late Miocene onwards with peak origins in late Pliocene; Bond, 2015) could have promoted its spread, as *C. purpureus s.l.* is also frequently found in fire-influenced habitats (Clément and Touffet, 1988; even called “fire moss” as a common name). Furthermore, in the recent post-quaternary period, the origin and expansion of urban environments provided major sources of anthropogenically influenced disturbance potentially favorable to *C. purpureus s.l.*

### Multiple Antarctic Colonizations, Including an Ancient Lineage

*C. purpureus s.l.* is one of the most widespread moss species found across the Antarctic (Ochyra et al., 2008). Ochyra et al. (2008) noted considerable diversity in leaf size and morphology between Antarctic *C. purpureus s.l.* plants, suggesting the presence of multiple lineages within Antarctica, a finding confirmed here by the presence of multiple origins of Antarctic populations (clades I, V, and VI). This reveals that Antarctica is not as isolated as is often assumed for spore-dispersed organisms (also seen in the Antarctic moss *Chorisodontium aciphyllum* (Hook. f. & Wils.) Broth.; Biersma et al., 2018a). Recently, Pisa et al. (2014) also proposed at least three independent origins of the moss *Bryum argenteum* Hedw. in Antarctica, and even the vascular plant *Colobanthus quitensis* (with much larger seeds than moss spores) was also found to have at least two independent origins in the Antarctic Peninsula (Biersma et al., 2020).

According to the dating analysis, Antarctic clade I, whose members consist only of specimens from the Antarctic Peninsula and the South Orkney Islands, has been isolated since the late Miocene or early Pliocene (**Table 1**; **Figures 5** and **S4**). Although more extensive sampling may be required to fully assess whether clade I is limited to Antarctica, its apparent ancient isolation suggests it may be a remnant lineage that has survived past glaciations in the maritime Antarctic *in situ*. Its arrival could have coincided with relatively warm interglacials on the Antarctic Peninsula during the early Pliocene (particularly between ~4.5–4.4 and ~3.6–3.4 Mya; De Schepper et al., 2014). Recent climate and glaciological modeling studies have highlighted greater dynamism in glacial extent than previously considered possible throughout the early Pliocene and Pleistocene (Scherer et al., 2008; Naish et al., 2009; Pollard and DeConto, 2009; De Schepper et al., 2014; DeConto and Pollard, 2016), suggesting the possibility of ice-free local refugial areas persisting throughout these periods (e.g. as suggested by Fraser et al., 2014). Molecular, phylogenetic and biogeographic studies also suggest *in situ* survival for many groups of terrestrial fauna in Antarctica throughout the Quaternary, Neogene and even Paleogene (see Convey et al., 2008, 2009, 2020, and references therein). Recently, increased evidence has also been found of million-year persistence of the Antarctic flora, e.g. several species of *Schistidium* (Biersma et al., 2018b), and *B. argenteum* (Pisa et al., 2014). Here, our data indicate that at least one lineage (I) of *C. purpureus s.l.* may also have had a long-term Antarctic presence *in situ*.

## Data Availability Statement

Sequences are uploaded to GenBank with accessions MN542517–MN542603, MN542604–MN542650, MN552307–MN552379 and MN556618–MN556666 (see **Supplementary Table S1**). Phylogenetic matrixes (MrBayes and BEAST input files), tree files and POPART input files are uploaded in the **Supplementary Material**.

## Author Contributions

PC, EB, RW, and SR conceived the study, with details further developed by JJ, MD, KL, and HG. SR, MD, and RW conceived and executed a small pilot study. EB, BV, and RW conducted the majority of herbarium sampling. EB and RW carried out the molecular work. EB, with guidance from JJ, conducted the analyses and wrote the manuscript. All authors contributed to the article and approved the submitted version.

## Funding

This study was funded by a Natural Environment Research Council (NERC) PhD studentship (ref NE/K50094X/1), NERC-CONICYT (NE/P003079/1) and Carlsberg Foundation (CF18-0267) to EB and NERC core funding to the BAS Biodiversity, Evolution, and Adaptation Team, and Australian Research Council (ARC) Discovery Project DP180100113 funding to SR and PC.

## Conflict of Interest

The authors declare that the research was conducted in the absence of any commercial or financial relationships that could be construed as a potential conflict of interest.
