# Supplementary material for: Latitudinal Biogeographic Structuring in the Globally Distributed Moss Ceratodon purpureus
Source: Front Plant Sci. 2020 Aug 28;11:502359. doi: 10.3389/fpls.2020.502359 (PMC7484499; doi:10.3389/fpls.2020.502359)
Supplement: Supplementary file 2 [file DataSheet_2.docx]

Supplementary Material

**Supplementary Table S1.** Sample list including herbarium and location details. Literature references of samples published before are given below. Samples originated from herbaria at the British Antarctic Survey (AAS), Botanic Garden, Meise (BR), British Museum of Natural History (BM), Royal Botanical Gardens Edinburgh (E), the University of Wollongong (WOLL) and the New York Botanical Garden (NY), augmented by fresh collections during expeditions of authors (EB, PC, SR).

| **Label in study** | **Geographic Origin** | **Ref.** | **Herbarium or University** | **Collection** | **Coordinates** | ***rps4*** | ***atpB-rbcL*** | ***trnL-F*** | ***ITS*** |
| --- | --- | --- | --- | --- | --- | --- | --- | --- | --- |
| AAS 03988 Ant. Peninsula | Recess Cove, Charlotte Bay, Ant. Peninsula |  | AAS | Smith R.I.L. 03988 | -64.58, -61.63 | MN542517 | MN542618 | MN552307 | MN556620 |
| AAS 07625 Ant. Peninsula | James Ross I., Ant. Peninsula |  | AAS | Smith R.I.L. 07625 | -63.87, -57.90 | MN542518 | MN542619 | MN552308 |  |
| CP24 S. Orkney Is. | Moe I, off Signy Is., South Orkney Is. | 1,2 | AAS | Smith R.I.L. 5237 | -60.44, -45.41 | MN542519 |  |  | MN556619 |
| BR 12045376 Papua New Guinea | Mt. Kaindi, Papua New Guinea |  | BR 5040120453763 | Hoffmann I. 90-414 | -7.33, 146.68 ^1^ | MN542520 | MN542610 | MN552309 | MN556631 |
| BR 32032632 Costa Rica | Serra de la Muerte, Costa Rica |  | BR 5040320326324 | Arts. T. CR 14/27 | 9.56, -83.80 ^1^ | MN542521 | MN542611 | MN552310 |  |
| BR 27280945 Bolivia | NW of Quime, Bolivia |  | BR 5040272809456 | Lewis M. 87585 | -16.95, -67.28 | MN542522 | MN542604 | MN552311 |  |
| AY881045 Reunion I. | Piton de la Fournaise, Reunion I. | 3 | DUKE | Arts REU 44/15 | -21.24, 55.71 ^1^ |  | AY881045 | | |
| BM 20823 Mexico | W of Popocatépetl, Sierra Nevada, Mexico |  | BM | Herman F.J. 20823 | 19.02, -98.63 ^1^ | MN542523 |  | MN552312 |  |
| CP17 Nepal | Sindure, Nepal | 1,2 | RBGE | Long D.G. 16417 | 28.17, 84.30 | MN542524 |  | MN552313 |  |
| BR 27272962 Greece | Chania, Omalos, Crete, Greece |  | BR 5040272729624 | Gradstein S.R. & Smittenberg J.H. 1214 | 35.34, 23.90 ^1^ | MN542525 | MN542625 | MN552314 | MN556618 |
| AY881059 Greece | Argalasti, Greece | 3 | DUKE | Cano & Ros 23.3.1999 | 39.23, 23.22 ^1^ |  | AY881059 | | |
| BM 27 Canary Is. | Above Aguamansa, Canary Is. |  | BM | Champion C.L. 27 | 28.37, -16.50 ^1^ | MN542526 |  | MN552315 |  |
| BM R08277a Reunion I. | Le Maido, W of Cirque de Mafate, Reunion I. |  | BM | Ellis L. & Wilbraham J. R08-277a | -21.40, 55.23 | MN542527 | MN542626 | MN552316 | MN556621 |
| CP13 Malawi | Lichenya, Malawi | 1,2 | RBGE | Longton R.E. M8593A | -15.55, 35.83 ^1^ | MN542528 | MN542627 | MN552317 |  |
| AY881052 Reunion I. | Piton de la Fournaise, Reunion I. | 3 | DUKE | Arts REU 44/15 | -21.24, 55.71 ^1^ |  | AY881052 | | |
| AY881057 South Africa | Table Mt, W Cape Prov., South Africa | 3 | DUKE | Hedderson 13371 | -33.96, 18.41 ^1^ |  | AY881057 | | |
| AB980065 Uganda | Rwenzori Mountains, Stanlay Plateau, Uganda |  | Uetake et al., unpubl. | Uetake J. (“taxon:3225”) | 0.38, 29.88 | AB848717 | AB980065 | | |
| AY881031 Indonesia | Amlapura, Bali, Indonesia | 3 | DUKE | Schäfer-Verwimp 21099 | -8.46, 115.61 ^1^ |  | AY881031 | | |
| AY881034 Chile | PN Alerces Osorno, Chile | 3 | DUKE | Cox 602/00 | -41.11, -72.36 ^1^ |  | AY881034 | | |
| AY881046 Wilkes sector, Antarctica | Casey Base, Antarctica | 3 | DUKE | Robinson & Wasley 99/00 | -66.28, 110.53 ^1^ |  | AY881046 | | |
| AY881048 Chile | Navarino I., Magellanes, Chile | 3 | DUKE | Goffinet 6990 | -55.11, -67.71 ^1^ |  | AY881048 | | |
| AY881053 Australia | Kangaroo I., S. Australia | 3 | NY | Streimann 54883 | -35.77, 137.21 ^1^ |  | AY881053 | | |
| AY881051 Tasmania, Australia | Hobart, Tasmania, Australia | 3 | DUKE | Newton & Bell 5780 | -42.88, 147.32 ^1^ |  | AY881051 | | |
| AY881049 Australia | Oberon, New South Wales, Australia | 3 | NY | Gilmore 65 | -33.70, 149.85 ^1^ |  | AY881049 | | |
| AY881043 Oregon, USA | Columbia, R. Gorge, Oregon, USA | 3 | DUKE | Sargent 6.VIII.00 | 45.71, -121.52 ^1^ |  | AY881043 | | |
| AY881037 California, USA | Pt. Reyes, NS California, USA | 3 | DUKE | Shaw 10 090 | 38.04, -122.80 ^1^ |  | AY881037 | | |
| AY881040 Australia | Stanthorpe, Queensland, Australia | 3 | NY | Streimann 52925 | -28.65, 151.94 ^1^ |  | AY881040 | | |
| AAS 03708 Ant. Peninsula | Joinville I., Ant. Peninsula |  | AAS | Smith R.I.L. 03708 | -63.25, -55.75 | MN542529 | MN542620 | MN552318 | MN556633 |
| AAS 04324 Ant. Peninsula | W of Lahille I., Ant. Peninsula |  | AAS | Smith R.I.L. 04324 | -65.53, -64.37 | MN542530 | MN542621 | MN552319 | MN556644 |
| BR 01907765 Idaho, USA | Boundary County, Copeland, Idaho, USA |  | BR 5040019077650 | Anderson L.E. 22618 | 48.90, -116.38 ^1^ | MN542531 | MN542622 | MN552320 | MN556630 |
| BM BM000977576 Montana, USA | Targhee Pass, W Yellowstone, Montana, USA |  | BM | Stern R.C. BM000977576 | 44.67, -111.28 ^1^ | MN542532 | MN542623 | MN552321 |  |
| BM 21661 Alaska, USA | Valdez Quadrangle, Old Valdez, Alaska, USA |  | BM | Herman F.J. 21661 | 61.11, -146.26 ^1^ | MN542533 | MN542624 | MN552322 | MN556653 |
| CP25 S. Shetland Is. | West Baily Head, Deception I., S. Shetland Is. | 1,2 | AAS | Mason D. 00001 | -62.58, -60.30 | MN542534 | MN542605 | MN552323 |  |
| CP32 Ant Peninsula | Charcot Is, Ant. Peninsula | 1,2 | AAS | Convey P. 00368 | -69.45, -75.15 | MN542535 | MN542612 | MN552324 |  |
| CP28 Ant. Peninsula | Danco Coast, Cuverville I, Ant. Peninsula | 1,2 | AAS | Leeuw C. de 00020 | -64.41, -62.38 | MN542536 | MN542613 | MN552325 | MN556645 |
| CP34 Ant. Peninsula | W Anchorage I., Ant. Peninsula | 1,2 | AAS | Smith R.I.L. 09212 | -67.36, -68.14 | MN542537 | MN542614 | MN552326 | MN556634 |
| CP30 S. Sandwich Is. | Irving Point, Visokoi I., S. Sandwich Is. | 1,2 | AAS | Convey P. 00242 | -56.43, -27.04 | MN542538 | MN542615 | MN552327 | MN556638 |
| AY881058 India | Lam Pokhari, Sikkim, India | 3 | DUKE | Long 22687 | 27.50, 88.20 ^1^ |  | AY881058 | | |
| AY881047 Illinois, USA | Schuyler, Illinois, USA | 3 | NY | Hill 30219 | 40.18, -90.63 ^1^ |  | AY881047 | | |
| AY881036 Alaska, USA | Denali N. P., Alaska, USA | 3 | DUKE | Vanderpoorten 4998 | 63.06, -151.00 ^1^ |  | AY881036 | | |
| BR 02840177 S. Shetland Is. | King George I., Admiralty Bay, S. Shetland Is. |  | BR 5040028401774 | Ochyra R. 4887/79 | -62.17, -58.44 | MN542539 |  | MN552328 |  |
| AAS 00064 S. Shetland Is. | Deception I., S. Shetland Is. |  | AAS | Smith R.I.L. 00064 | -63.00, -60.52 | MN542540 |  |  |  |
| CP23 S. Shetland Is. | Pendulum Cove, Deception I, S. Shetland Is. | 1,2 | AAS | Smith R.I.L. 11191 | -62.56, -60.36 | MN542541 |  | MN552329 | MN556635 |
| CS30 S. Shetland Is. | Carlini Station, King George I., S. Shetland Is. |  | WOLL | Casanova-Katny A. CS30 | -62.23, -58.67 | MN542542 |  |  |  |
| AAS 725 Argentina | Argentina |  | AAS ACHE | Matteri-Schiavone 725 | -54.50, -68.00 | MN542543 | MN542628 | MN552330 |  |
| AAS 4545909 Wilkes sector, Antarctica | Clark Pen, Wilkes Sector, Antarctica |  | AAS ACHE | Seppelt R.D. 5909 ACHE 454 | -66.15, 110.36 | MN542544 | MN542629 | MN552331 |  |
| AAS 04120 Ant. Peninsula | Omega I., Melchior I., Ant. Peninsula |  | AAS | Smith, R.I.L. 04120 | -64.33, -62.93 | MN542545 | MN542630 | MN552332 |  |
| AAS 09644A Ross sector, Antarctica | Harrow Peaks, Victoria Land. |  | AAS | Smith, R.I.L. 09644A | -74.07, 164.75 | MN542546 | MN542631 | MN552333 | MN556627 |
| AAS 00165 S. Orkney Is. | W of Matthews I., S. Orkney Is. |  | AAS | Smith, R.I.L. 00165 | -60.75, -45.17 | MN542547 | MN542632 | MN552334 | MN556636 |
| Biersma 18 Chile | Navarino I., Magellanes, Chile |  | BAS collections | Biersma, E.M. 18 | -54.93, -67.28 ^1^ | MN542548 | MN542633 | MN552335 | MN556632 |
| BM 33207 Chile | 5 km E of Quidico, Prov. Arauco, Chile |  | BM | Ireland, R.R. & Bellolio, G. 33207 | -38.15, -73.27 | MN542549 | MN542634 | MN552336 |  |
| CP5 Australia | Mt Beauty, Victoria, Australia | 1,2 | WOLL ARC | Clarke, L.J. CP5 | -36.74, 147.17 | MN542550 | MN542635 | MN552337 | MN556625* MN556650* |
| CP44 Heard I. | Dovers Moraine, Heard I. | 1,2 | WOLL ARC | Turnbull, J.D. CP44 | -53.12, 73.70 | MN542551 | MN542607 | MN552338 | MN556651 |
| CP21 Maud Land, Antarctica | Ice Axe Peak, Maud Land, Antarctica | 1,2 | AAS | Watkins, B.P. 00020A | -71.47, -3.25 | MN542552 | MN542608 | MN552339 | MN556642 |
| CP39 Heard I. | Paddock Valley, Heard I. | 1,2 | WOLL ARC | Turnbull, J.D. CP39 | -53.08, 73.50 | MN542553 | MN542609 | MN552340 |  |
| BR 02155925 Switzerland | Valais, Bagnes, Versegers, Switzerland |  | BR 5040021559250 | Lawalrée, A. 23282 | 46.06, 7.23 ^1^ | MN542554 | MN542636 | MN552341 | MN556647 |
| BR 02316781 Switzerland | Valais, Bagnes, Lourtier, Switzerland |  | BR 5040023167811 | Lawalrée, A. 24147 | 46.04, 7.26 ^1^ | MN542555 | MN542637 | MN552342 | MN556654 |
| BR 08930464 Poland | Plaine de Walcz, Ilowiec, Poland |  | BR 5040089304649 | Lisowski, S., Rusinska, A. & Melosik, I. 27 | 50.83, 23.40 ^1^ | MN542556 | MN542638 |  |  |
| BR 12018401 Siberia, Russia | Baikal lake, Listvianka, Siberia, Russia |  | BR 5040120184018 | Vašák, V. s.n. | 51.85, 104.83 ^1^ | MN542557 | MN542639 | MN552343 |  |
| BR 13051247 Svalbard | Isbjörnkamna, Honsund, Svalbard |  | BR 5040130512474 | Godzik, B. & Grodzinska, K. s.n. | 77.02, 15.57 | MN542558 | MN542616 | MN552344 | MN556655 |
| BR 13622234 France | Foret de Fontainebleau, France |  | BR 5040136222346 | Bamps, P. 4685 | 48.41, 2.63 ^1^ | MN542559 | MN542640 | MN552345 |  |
| BR 30658767 Japan | Campus Hiroshima University, Japan |  | BR 5040306587671 | Yamaguchi, T. & Iwatsuki, Z. 29 | 36.07, 138.08 ^1^ | MN542560 | MN542641 | MN552346 |  |
| BR 31255218 Hawaii, USA | Near Pu'u hulu-hulu, Big Island, Hawaii, USA |  | BR 5040312552182 | Arts, T. HAW 11/04 | 19.37, -155.20 ^1^ | MN542561 | MN542642 | MN552347 |  |
| BR 31460231 Ecuador | Imbabura, Lago Cuicocha, Ecuador |  | BR 5040314602311 | Arts, T. 13/007A | 0.38, -78.11 ^1^ | MN542562 | MN542617 | MN552348 |  |
| BR 36261428 Vermont, USA | Chittenden, Vermont, USA |  | BR 5040362614281 | White, H. & White, C. 1158 | 43.73, -72.94 ^1^ | MN542563 | MN542643 | MN552349 | MN556656 |
| BR 27271447 Hawaii, USA | Oahu, Kaala, Hawaii, USA |  | BR 5040272714477 | Hoe W.J. 3048.0 | 21.44, -158.00 ^1^ | MN542564 | MN542644 | MN552350 | MN556657 |
| BR 27272356 Quebec, Canada | Iles-de-la-Madeleine, Quebec, Canada |  | BR 5040272723561 | De Sloover J.L. 7786 | 47.38, -61.88 ^1^ | MN542565 | MN542645 | MN552351 | MN556662 |
| BR 27270639 Colorado, USA | Clear Creek County, Colorado, USA |  | BR 5040272706397 | Miller J.S. 6887 | 39.82, 105.75 | MN542566 | MN542606 | MN552352 |  |
| CP8 Hawaii, USA | Mauna Kea, Hawaii, USA | 1,2 | RBGE | Degener O. & Degener I. 34420 | 19.82, -155.47 | MN542567 | MN542646 | MN552353 | MN556622* MN556663* |
| CP11 New York, USA | New York (Botanical Gardens), USA | 1,2 | NY | S.N. | 40.86, -73.88 | MN542568 | MN542647 | MN552354 | MN556658 |
| BM A1003 Alaska, USA | Chandler Lake, Endicott Mts, Alaska, USA |  | BM | Smith, G.L. A1003 | 68.12, -152.47 | MN542569 | MN542648 | MN552355 | MN556643 |
| BM 1228 Japan | Honshu, Tsukuba-shi, Amakubo, Japan |  | BM | Higuchi, M. 1228 | 36.06, 140.06 | MN542570 | MN542649 | MN552356 |  |
| BR 02873422 UK | Bedfordshire, UK |  | BR 5040028734223 | Jury, S.L. J2359 | 51.92, 0.66 | MN542571 | MN542650 | MN552357 |  |
| AY881062 Morocco | Bab Bou Idir, Morocco | 3 | DUKE | Cano and Ros 21.4. 1997 | 34.07, -4.12 ^1^ |  | AY881062 | | |
| AY881061 Arizona, USA | Madira Canyon, Arizona, USA | 3 | NY | Schofield 105061 | 34.05, -111.09 ^1^ |  | AY881061 | | |
| AY881044 Ecuador | Otavalo, Ecuador | 3 | DUKE | McDaniel E112 | 0.23, -78.26 ^1^ |  | AY881044 | | |
| AY881038 Colorado, USA | Boulder, Colorado, USA | 3 | DUKE | Weber 19426 | 40.01, -105.27 ^1^ |  | AY881038 | | |
| AY881060 New York, USA | Adirondack Mts, New York, USA | 3 | NY | Miller 11911 | 43.95, -73.73 ^1^ |  | AY881060 | | |
| AY881055 Germany | Baden-Württemberg, Germany | 3 | DUKE | Schäfer-Verwimp 25550 | 48.66, 9.35 ^1^ |  | AY881055 | | |
| AY881054 Alberta, Canada | Edson, Alberta, Canada | 3 | NY | Schofield 103437 | 53.58, -116.44 ^1^ |  | AY881054 | | |
| AY881050 Siberia, Russia | Kurgan, Siberia, Russia | 3 | DUKE | Kurbatova 21 V 2000 | 55.46, 65.30 ^1^ |  | AY881050 | | |
| AY881042 New York, USA | Ithaca, New York, USA | 3 | DUKE | Shaw 12.VI. 2000 | 42.44, -76.50 ^1^ |  | AY881042 | | |
| AY881041 Sweden | Hamra, Halsingland, Sweden | 3 | S | Hedenäs B38313 | 61.66, 15.01 ^1^ |  | AY881041 | | |
| AY881039 Liaoning, China | Baishilaji Reserve, Liaoning, China | 3 | NY | Buck 23783 | 41.83, 123.43 ^1^ |  | AY881039 | | |
| AY881035 Nova Scotia, Canada | Digby, Nova Scotia, Canada | 3 | NY | Schofield 95576 | 44.62, -65.76 ^1^ |  | AY881035 | | |
| AY881033 France | Soultzeren, Alsace, France | 3 | DUKE | Untereiner 12.05.00 | 48.06, 7.10 ^1^ |  | AY881033 | | |
| AY881032 Germany | Baden-Württemberg, Germany | 3 | DUKE | Schäfer-Verwimp 25550 | 48.66, 9.35 ^1^ |  | AY881032 | | |
| AY881056 Pennsylvania, USA | Palmerton, Pennsylvania, USA | 3 | DUKE | McDaniel B135 | 40.80, -75.61 ^1^ |  | AY881056 | | |
| Biersma 21 Chile | Navarino I., Magellanes, Chile |  | BAS collections | Biersma, E.M. 21 | -54.95, -67.26 ^1^ | MN542572 |  | MN552358 | MN556649 |
| BM 31149 Chile | 6 km S of Contulmo, Prov. Arauco, Chile |  | BM | Ireland, R.R. & Bellolio, G. 31149 | -38.00, -73.13 | MN542573 |  | MN552359 | MN556648 |
| BM 33951 Chile | Waterfalls near Santa Juana, S of Curali, Chile |  | BM | Ireland, R.R. & Bellolio, G. 33951 | -37.15, -72.57 | MN542574 |  | MN552360 |  |
| CP16 Australia | Australian Nat. Uni., Canberra, Australia | 2 | WOLL ARC | Clarke, L.J. CP16 | -35.28, 149.12 | MN542575 |  | MN552361 |  |
| 219 Wilkes sector, Antarctica | Clark Peninsula, Antarctica | 2,4 | WOLL ARC | Robinson, S.A. 219 | -66.25, 110.57 | MN542576 |  | MN552362 | MN556626 |
| Biersma 1 Chile | Navarino I., Magellanes, Chile |  | BAS collections | Biersma, E.M. 1 | -54.93, -67.60 ^1^ | MN542577 |  |  | MN556666 |
| Biersma 4 Chile | Navarino I., Magellanes, Chile |  | BAS collections | Biersma, E.M. 4 | -54.93, -67.61 ^1^ | MN542578 |  |  | MN556624 |
| Biersma 20 Chile | Navarino I., Magellanes, Chile |  | BAS collections | Biersma, E.M. 20 | -54.95, -67.21 ^1^ | MN542579 |  |  |  |
| BR 27264979 Australia | Leura Gap, Bimberi Range, Canberra, Australia |  | BR 5040272649793 | Streimann H. 34959 | -35.63, 148.77 | MN542580 |  |  |  |
| CP6 Wilkes sector, Antarctica | ASPA135 Melt Lake, Windmill Is., Antarctica | 2,4 | WOLL ARC | Robinson, S.A. CP6 | -66.33, 110.47 ^1^ | MN542581 |  | MN583240 |  |
| CP7 Australia | Macquarie University, Ryde, Sydney, Australia | 2 | WOLL ARC | Downing, A. CP7 | -33.77, 151.11 | MN542582 |  |  |  |
| 76 Wilkes sector, Antarctica | Red Shed, Casey, Windmill Is., Antarctica | 2,4,10 | WOLL ARC | Robinson, S.A. 76 | -66.28, 110.53 | MN542583 |  |  |  |
| CPMP Wilkes sector, Antarctica | ASPA135 A2, Windmill Is., Antarctica | 2,4,10 | WOLL ARC | Robinson, S.A. CPMP | -66.28, 110.54 | MN542584 |  |  |  |
| WPD8 Wilkes sector, Antarctica | ASPA136 C1, Windmill Is., Antarctica | 2,4,10 | WOLL ARC | Robinson, S.A. WPD8 | -66.25, 110.56 | MN542585 |  |  |  |
| B10 Australia | Wollongong, Australia | 2 | WOLL ARC | Wyber, R. B10 | -34.40, 150.86 | MN542586 |  |  |  |
| B11 Australia | Wollongong, Australia | 2 | WOLL ARC | Wyber, R. B11 | -34.40, 150.86 | MN542587 |  |  |  |
| B12 Australia | Wollongong, Australia | 2 | WOLL ARC | Wyber, R. B12 | -34.40, 150.86 | MN542588 |  |  |  |
| B20 Wilkes sector, Antarctica | Casey Station, Windmill Is., Antarctica | 2,4 | WOLL ARC | Robinson, S.A. B20 | -66.28, 110.69 | MN542589 |  |  | MN556623 |
| WPD5 Wilkes sector, Antarctica | ASPA136 C1, Windmill Is., Antarctica | 2,4,10 | WOLL ARC | Robinson, S.A. WPD5 | -66.25, 110.56 | MN542590 |  |  | MN556639 |
| 166CP Wilkes sector, Antarctica | ASPA135, Melt Lake, Windmill Is., Antarctica | 2,4 | WOLL ARC | Robinson, S.A. 166CP | -66.28, 110.54 | MN542591 |  |  |  |
| Biersma 2 Chile | Navarino I., Magellanes, Chile |  | BAS collections | Biersma, E.M. 2 | -54.93, -67.60 ^1^ | MN542592 |  |  |  |
| CP20 Victoria Land, Antarctica | Edmonson Pnt, Victoria Land, Antarctica | 1,2 | AAS | Smith R.I.L. S.N. | -74.20, 165.08 | MN542593 |  | MN552363 |  |
| AJ554004 UK | Berkshire, Reading, UK | 5 | RNG | S.N. | 51.45, -0.98 ^1^ | AJ554004 | | |  |
| BR 01726701 Wisconsin, USA | St Croix Falls, Wisconsin, USA |  | BR 5040017267015 | Janssens, J.A. 8222 | 45.41, -92.64 ^1^ | MN542594 |  | MN552364 |  |
| BR 31381116 Spain | Erjos, near the Casa Forestal, Tenerife |  | BR 5040313811165 | Arts, T. 24078 | 28.29, -16.62 ^1^ | MN542595 |  | MN552365 | MN556659 |
| BR 33783783 Luxemburg | Consdorf, Kalkesbaach, Luxemburg |  | BR 5040337837837 | Arts, T. 15180 | 49.82, 6.35 ^1^ | MN542596 |  | MN552366 |  |
| BM 14349 British Colombia, Canada | Howe Sound, British Columbia, Canada |  | BM | Schofield, W.B. 14349 | 49.15, 123.15 | MN542597 |  | MN552367 | MN556664 |
| BR 01997691 Poland | Sejny Lake District, Giby-Podkaczan, Poland |  | BR 5040019976915 | Ochyra R. 220 | 54.11, 23.35 ^1^ | MN542598 |  |  | MN556660 |
| BR 33783581 Switzerland | Luzern, Gütschenwald, Switzerland |  | BR 5040337835819 | Arts, T. 1218 | 47.05, 8.28 ^1^ | MN542599 |  |  | MN556646 |
| BR 11900583 Siberia, Russia | Pikhtovaia gora, Bratsk, Siberia, Russia |  | BR 5040119005836 | Vašák, V. s.n. | 56.17, 101.60 ^1^ | MN542600 |  |  | MN556652 |
| BR 01997893 Cuacasus, Russia | Itkol, Baksan, Mnt. Elbrus, Caucasus |  | BR 5040019978933 | Vašák, V. s.n. | 43.68, 43.54 ^1^ | MN542601 |  |  | MN556665 |
| BR 16775643 British Columbia, Canada | SE of Chilliwack Lake, British Columbia, Canada |  | BR 5040167756438 | Schofield, W.B. 57917 | 49.13, 121.23 | MN542602 |  |  |  |
| BR 31460433 Ecuador | Volcan Cotopaxi, near Sindipamba, Ecuador |  | BR 5040314604339 | Arts, T. 23/026A | -0.68, -78.43 ^1^ | MN542603 |  |  |  |
| AF435271 British Columbia, Canada | Bennett Bay, Mayne I., British Columbia, Canada | 6 | DUKE | Belland & Schofield 17530 | 48.84, -123.24 | AF435271 | | |  |
| FJ572605 NE China | Changbai, NE China | 7 | SHNU CB158 | S.N. | 42.00, 128.00 | FJ572605 | | |  |
| FJ572589 NE China | Changbai, NE China | 7 | SHNU CB43 | S.N. | 42.00, 128.00 | FJ572589 | | |  |
| AY908122 UK | St. Abbs, Scotland, UK | 8 | DUKE | Long, D.L. 21805 | 55.89, -2.14 ^1^ | AY908122 | | |  |
| AY908121 W Virginia, USA | Larenim County Park, W Virginia, USA | 8 | DUKE | Bachmann 538 | 38.59, -80.45 ^1^ | AY908121 | | |  |
| AAS 06209B Wilkes sector, Antarctica | E of Wilkes Station, Clark Peninsula, Antarctica |  | AAS | Smith, R.I.L. 06209B | -66.25, 110.55 |  |  | MN552368 | MN556640 |
| AAS 06213A Wilkes sector, Antarctica | E of Wilkes Station, Clark Peninsula, Antarctica |  | AAS | Smith, R.I.L. 06213A | -66.25, 110.55 |  |  | MN552369 | MN556641 |
| BR 33783278 Madeira | Madeira, Boca de Encumeade |  | BR 5040337832788 | Arts, T. 15982 | 32.76, -16.96 ^1^ |  |  | MN552370 |  |
| AAS 363835 New Zealand | Doubtful Sound, Fjordland, New Zealand |  | AAS CHR | Allan, H.H. 363835 | -45.43, 167.72 ^1^ |  |  | MN552371 |  |
| AAS 02192 Enderby sector Antarctica | West Ongul Island. |  | AAS | Shimizu, H. 02192 | -69.02, 39.53 |  |  | MN552372 |  |
| AAS 05301A Ant. Peninsula | Dundee I., Trinity Peninsula, Ant. Peninsula |  | AAS | Booth, R.G. 05301A | -63.48, -56.25 |  |  | MN552373 |  |
| AAS BA 02827 Chile | Puerto Toro, Prov. Magallanes, Chile |  | AAS BA | Matteri, C.M. 02827 | -51.40, -73.07 |  |  | MN552374 |  |
| CP02 Australia | Lucas Heights, Australia |  | WOLL ARC | Clarke, L.J. CP02 | -34.05, 150.98 |  |  | MN552375 |  |
| AAS 03231 S. Orkney Is. | Coronation I., S. Orkney Is. |  | AAS | Smith, R.I.L. 03231 | -60.67, -45.60 |  |  | MN552376 |  |
| BR 10334136 France | Aude, Pic de Nore, France |  | BR 5040103341360 | De Sloover J.L. 45210 | 43.42, 2.46 ^1^ |  |  | MN552377 | MN556629 |
| AAS 00139 Chile | S shore Lago Porteno, Prov. Magallanes, Chile |  | BA AAS | Greene, S.W. 00139 | -51.37, -72.75 |  |  | MN552378 |  |
| AAS 09876A Victoria Land, Antarctica | Harrow Peaks, Victoria Land, Antarctica |  | AAS | Smith, R.I.L. 09876A | -74.07, 164.75 |  |  | MN552379 | MN556628 |
| AY156591 Victoria Land, Antarctica | Edmonson Point, N. Victoria Land | 9 | - | S.N. | -74.03, 165.00 |  |  |  | AY156591 |
| AY613336 Victoria Land, Antarctica | Crater Cirque, N. Victoria Land | 9 | - | S.N. | -72.62, 169.37 |  |  |  | AY613336 |
| AY156589 Macquarie I. | Macquarie I. | 9 | - | S.N. | -54.62, 158.85 |  |  |  | AY156589 |
| AY156590 Victoria Land, Antarctica | Botany Bay, Victoria Land, Antarctica | 9 | - | S.N. | -76.88, 162.73 |  |  |  | AY156590 |
| AY156587 Australia | Sydney, Australia | 9 | - | S.N. | -33.87, 151.20 |  |  |  | AY156587 |
| AY156586 Australia | Canberra, Australia | 9 | - | S.N. | -35.30, 149.13 |  |  |  | AY156586 |
| AAS 00070A S. Shetland Is. | Deception I., S. Shetland Is. |  | AAS | Smith, R.I.L. 00070A | -63.00, -60.57 |  |  |  | MN556637 |
| BR 30659878 Italy | Sardegna, Broncu Spina, Gennargentu, Italy |  | BR 5040306598783 | Pedrotti, C.C. s.n. | 40.12, 9.01 ^1^ |  |  |  | MN556661 |
| AY156588 Heard I. | Heard I. | 9 | - | S.N. | -53.10, 73.52 |  |  |  | AY156588 |

^1^ = Longitudes and latitudes not provided with sample. Approximate location found via <http://mynasadata.larc.nasa.gov/latitudelongitude-finder/>

*= Multiple copies of ITS derived from the same specimens (in samples from Hawaii and Australia).
Samples or GenBank sequences used from references: 1: Clarke et al., 2008, 2: Wyber, 2013, 3: McDaniel and Shaw, 2005, 4: Clarke et al., 2009, 5: Hedderson et al., 2004, 6: La Farge et al., 2002, 7: Liu et al., 2010, 8: Shaw et al., 2005, 9: Skotnicki et al., 2004, 10: Robinson et al., 2018.

**References:**

Clarke, L. J., Ayre, D. J., and Robinson, S. A. (2008). Somatic mutation and the Antarctic ozone hole. *J. Ecol.* 96, 378–385. https://doi.org/10.1111/j.1365-2745.2007.01347.x

Clarke, L. J., Robinson, S. A., and Ayre, D. J. (2009). Genetic structure of Antarctic populations of the moss Ceratodon purpureus. *Antarct. Sci.* 21, 51–58. doi: https://doi.org/10.1017/S0954102008001466

Excoffier, L., and Lischer, H. E. (2010). Arlequin suite ver 3.5: a new series of programs to perform population genetics analyses under Linux and Windows. *Mol. Ecol. Resour.* 10, 564–567. doi: 10.1111/j.1755-0998.2010.02847.x

Hedderson, T. A., Murray, D. J., Cox, C. J., and Nowell, T. L. (2004). Phylogenetic relationships of haplolepideous mosses (Dicranidae) inferred from rps4 gene sequences. *Syst. Biol.* 29, 29–41. https://doi.org/10.1600/036364404772973960

La Farge, C., Shaw, A. J. and Vitt, D. H. (2002) The circumscription of the Dicranaceae (Bryopsida) based on the chloroplast regions trnL-trnF and rps4. *Syst. Bot.* 27, 435–452. https://doi.org/10.1043/0363-6445-27.3.435

Liu, Y., Yan, H. -F., Cao, T. and Ge, X. -J. (2010). Evaluation of 10 plant barcodes in Bryophyta (Mosses). *J. Syst. Evol.* 48, 36-46. https://doi.org/10.1111/j.1759-6831.2009.00063.x

McDaniel, S. F., and Shaw, A. J. (2005). Selective sweeps and intercontinental migration in the cosmopolitan moss Ceratodon purpureus (Hedw.) Brid. *Mol. Ecol.* 14, 1121–32. doi: https://doi.org/10.1111/j.1365-294X.2005.02484.x

Robinson, S. A., King, D. H., Bramley-Alves, J., Waterman, M. J., Ashcroft, M. B., Wasley, J., et al. (2018). Rapid change in East Antarctic terrestrial vegetation in response to regional drying. *Nat. Clim. Change* 8, 879–884. https://doi.org/10.1038/s41558-018-0280-0

Schofield, W.B. (1992). "Bryophyte distribution patterns." in Bryophytes and Lichens in a Changing Environment, ed. J.W. Bates and A.M. Farmer. (Oxford: Clarendon Press)

Shaw, A .J., Cox, C. J. and Goffinet, B. (2005). Global patterns of moss diversity: taxonomic and molecular inferences. *Taxon*, 54, 337-352. https://doi.org/10.2307/25065362

Skotnicki, M. L., Mackenzie, A. M., Ninham, J.A. and Selkirk, P.M. (2004) High levels of genetic variability in the moss *Ceratodon purpureus* from continental Antarctica, subantarctic Heard and Macquarie Islands, and Australasia. *Polar Biol.* 27, 687-698. https://doi.org/10.1007/s00300-004-0640-2

Wyber, R. A. (2013). Phylogeny of Three East Antarctic Mosses. [PhD thesis]. [Wollongong, (Australia)]: University of Wollongong

**
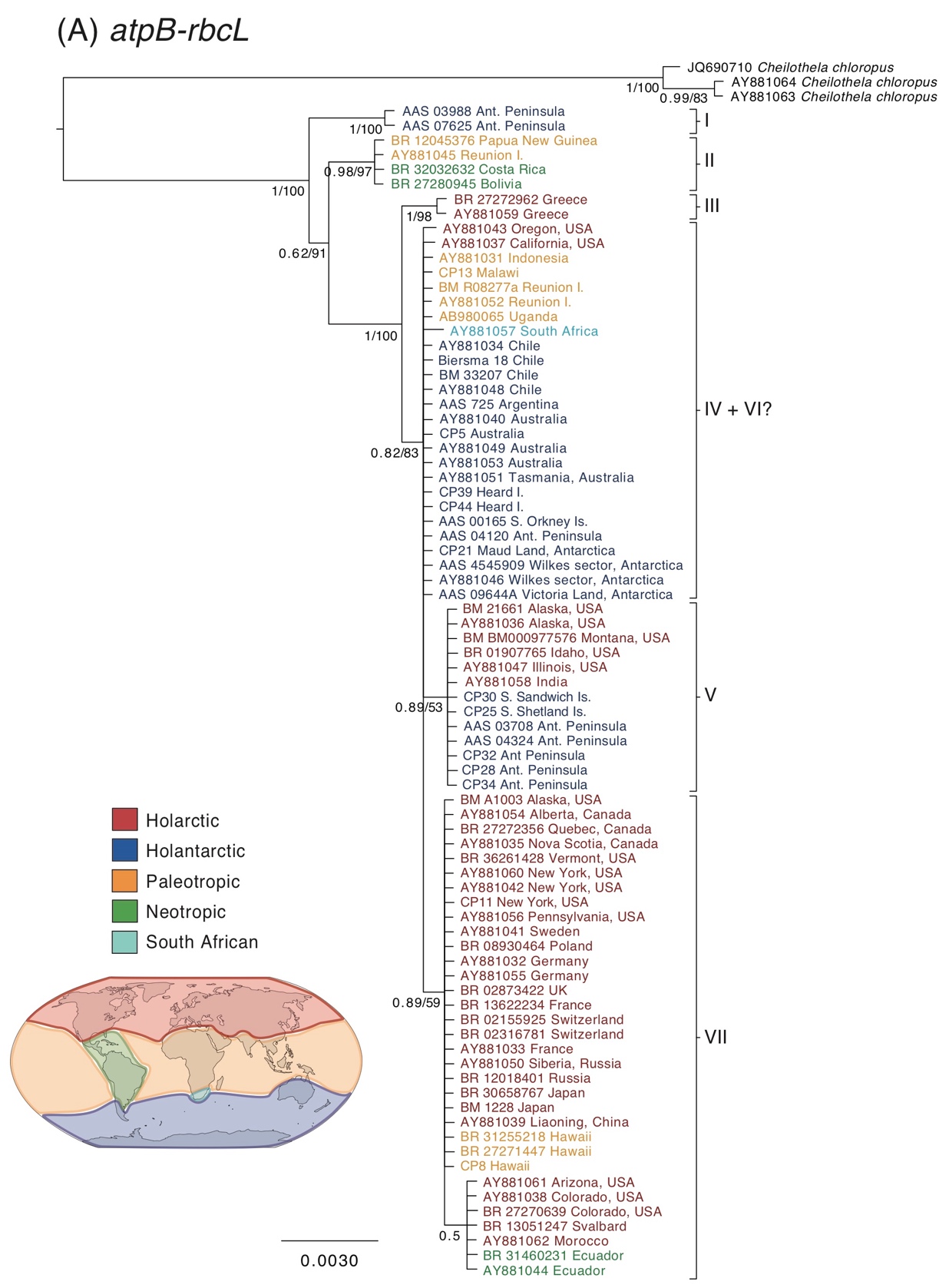
**

**Supplementary Fig. S1.** Bayesian phylogenies for *Ceratodon purpureus* constructed with cpDNA markers (A) *atpB-rbcL*, (B) *rps4*, (C) *trnL-F* and (D) a concatenated dataset of *atpB-rbcL* and *rps4* (for B-D see continuation in the next three pages). Taxon colours refer to the bryofloristic kingdoms of the world (see map; redrawn from Schofield, 1992) and do not follow the same colour scheme as Fig. 2 and 3, as singe gene trees do not show evidence of all clades. Outgroups are shown in black. The scale bar represents the mean number of nucleotide substitutions per site.


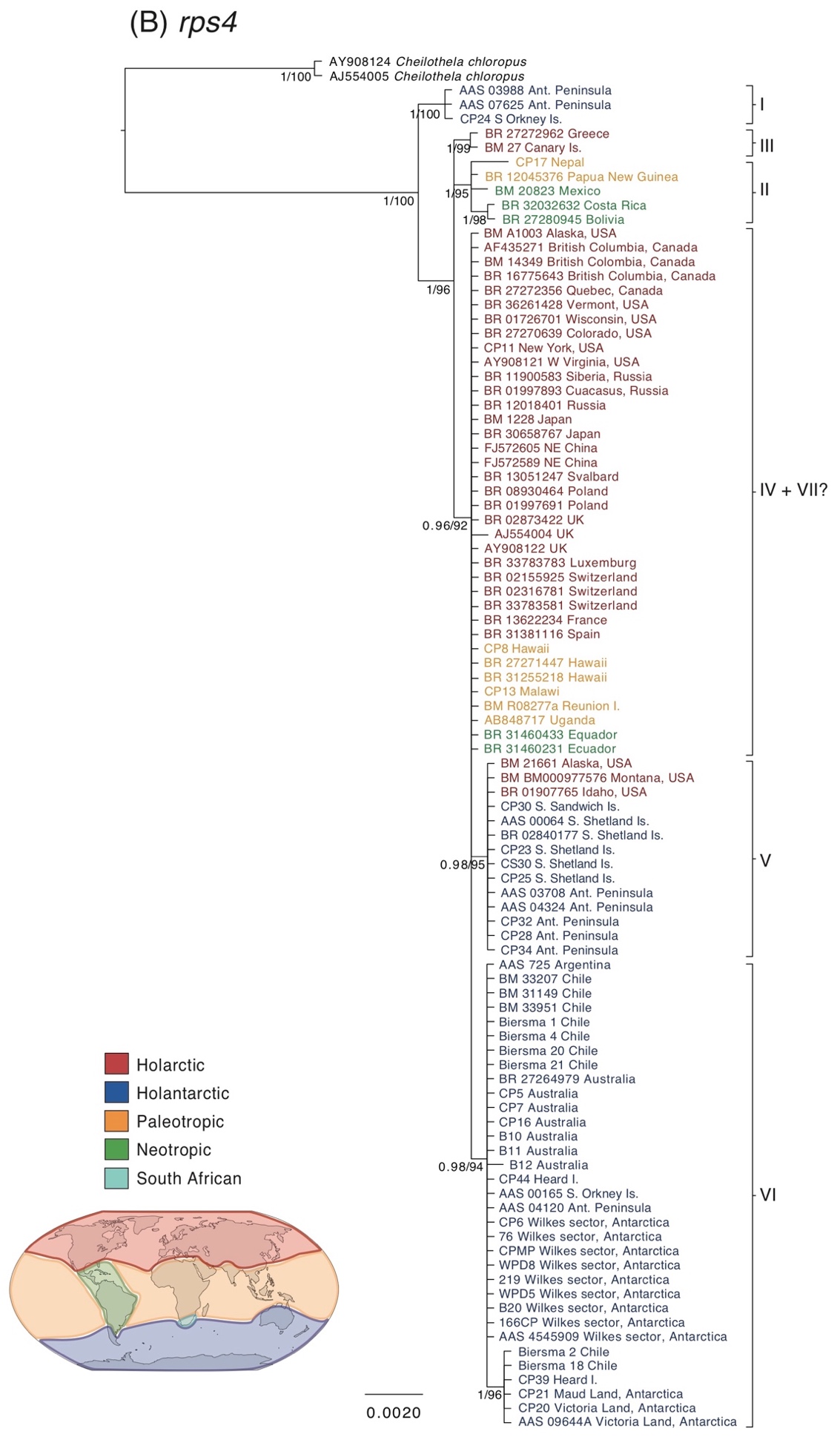


**Fig. S1.** (continued)

**
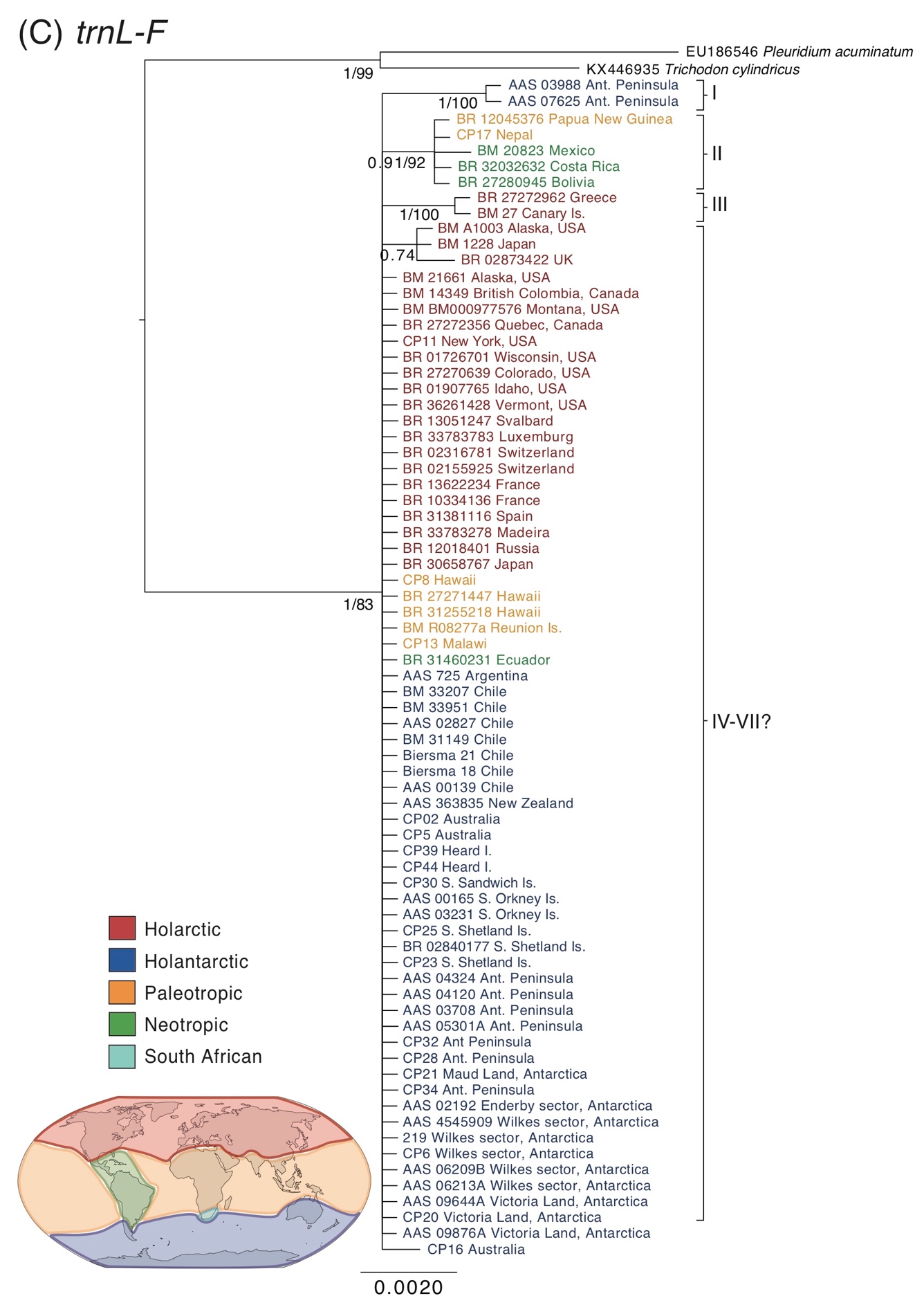
**

**Fig. S1.** (continued)

**
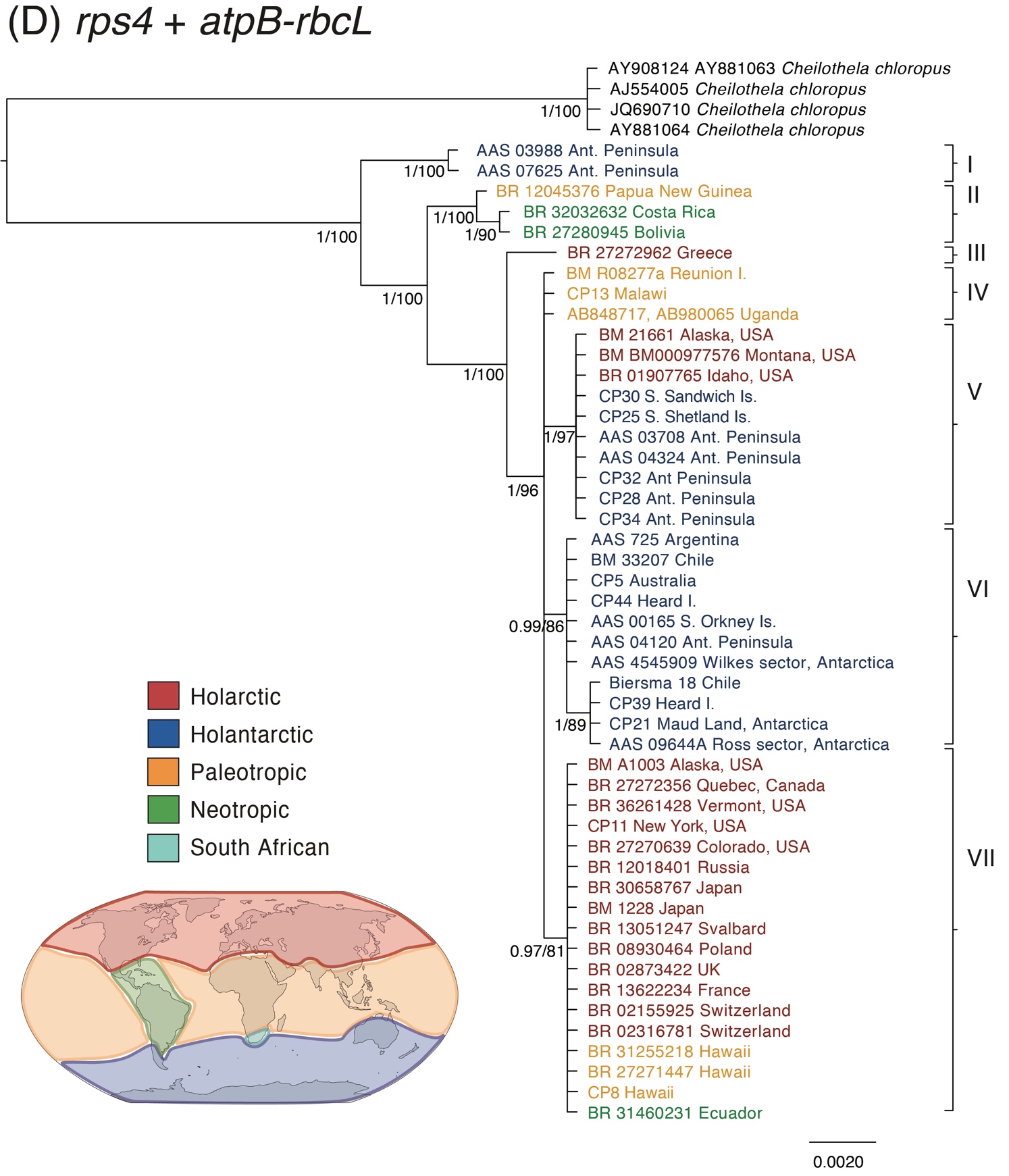
**

**Fig. S1.** (continued)


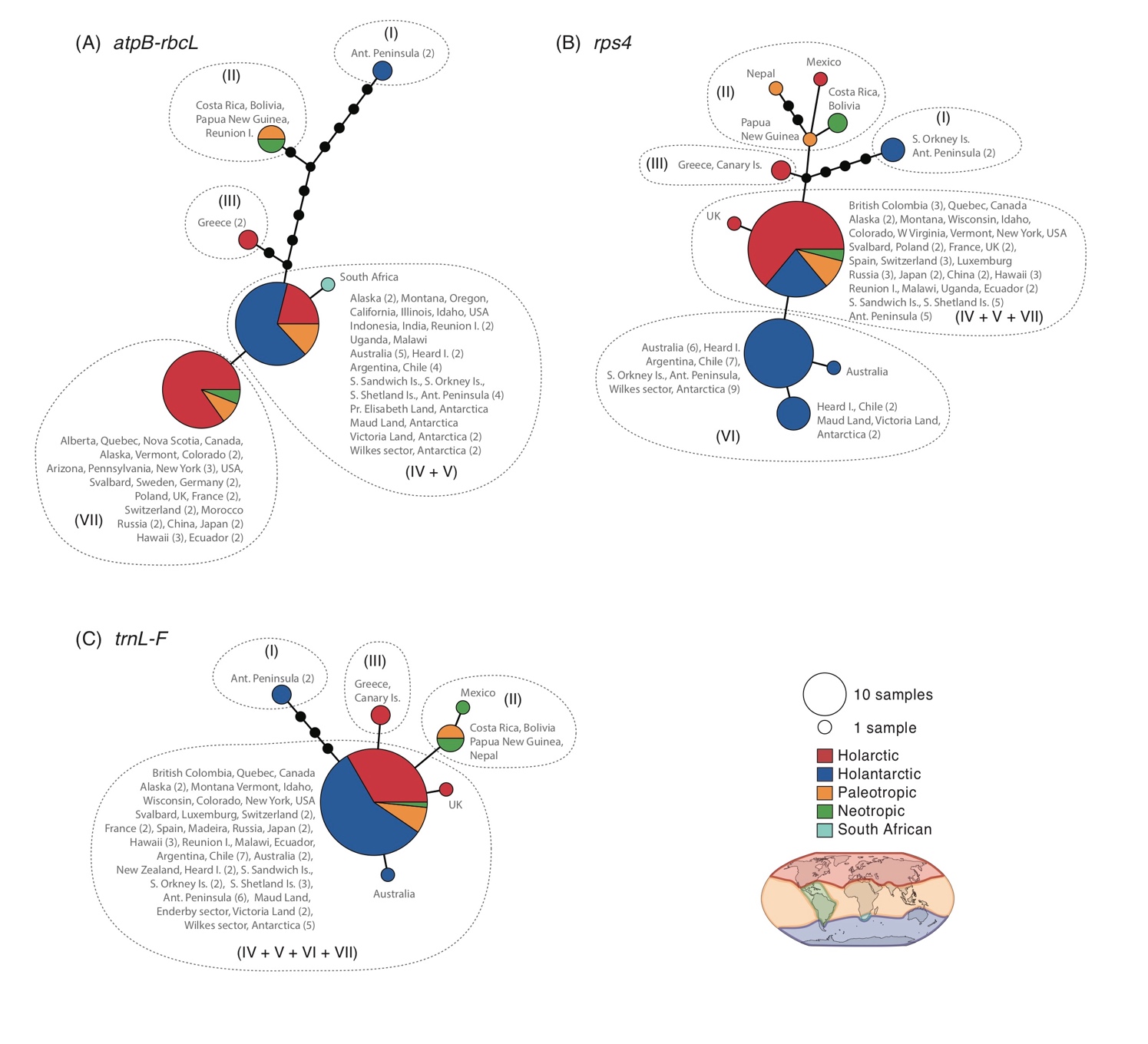
 **Supplementary Fig. S2.** Haplotype networks of cpDNA markers (A) *atpB-rbcL* (B) *rps4* and (C) *trnL-F* within the species *Ceratodon purpureus* with geographical information. Haplotype circle sizes correspond to the number of specimens. Colours refer to the bryofloristic kingdoms of the world (see map; redrawn from Schofield, 1992) and do not follow the same colour scheme as Figs. 2 and 3, as singe loci do not show evidence of all clades. Branches represent mutations between haplotypes, with mutations shown as 1-step edges.

| **Supplementary Table S2.** Genetic diversity indices, demographic and spatial expansion model tests and Tajima’s D and Fu’s Fs neutrality tests as performed in using ARLEQUIN v3.5.1.2 (Excoffier & Lischer, 2010) for *atpB-rbcL*, *rps4* and *trnL-F* within the species *Ceratodon purpureus.* Analyses were performed on total individual cpDNA loci as well as the concatenated cpDNA dataset. Within the latter, analyses were performed on all clades (I-VII; n=47) and the large ABGD-inferred cluster as defined with P_max_=0.0017-0.0046 (III-VII; n=42) and P_max_=0.001; (IV-VII; n=41), as shown in Fig. 2. SSD or HRI p-values were non-significant except where indicated with *. | | | | | | | | | | | | |  |
| --- | --- | --- | --- | --- | --- | --- | --- | --- | --- | --- | --- | --- | --- |
|  |  |  |  |  |  |  | Demographic expansion | | Spatial expansion | | Neutrality tests: |  | |
| Clades | n | bp^a^ | π | | S | *h* | SSD | HRI | SSD | HRI | Tajima's D (*P*) | Fu's Fs (*P*) | |
| *atpB-rbcL* (total) | 80 | 629 | 0.006±0.003 | | 17 | 0.793±0.026 | 0.047 | 0.092 | 0.043 | 0.092 | -1.248 (0.092) | 2.420 (0.835) | |
| *rps4* (total) | 94 | 589 | 0.003±0.002 | | 18 | 0.767±0.029 | 0.007 | 0.073 | 0.007* | 0.073 | -1.525 (0.038*) | -3.290 (0.089) | |
| *trnL-F* (total) | 74 | 435 | 0.002±0.002 | | 10 | 0.319±0.070 | 0.026 | 0.385 | 0.003 | 0.385 | -1.986 (0.002*) | -2.939 (0.066) | |
| Concatenated cpDNA |  |  |  | |  |  |  |  |  |  |  |  | |
| I-VII | 47 | 1660 | 0.004±0.002 | | 37 | 0.874±0.029 | 0.827* | 0.057 | 0.028 | 0.057 | -1.452 (0.050*)^1^ | 2.094 (0.809) | |
| III-VII | 42 | 1658 | 0.002±0.001 | | 11 | 0.843±0.033 | 0.035 | 0.083 | 0.037 | 0.083 | -0.873 (0.210) | 0.972 (0.708) | |
| IV-VII | 41 | 1657 | 0.002±0.001 | | 5 | 0.835±0.033 | 0.038 | 0.091 | 0.040 | 0.091 | 0.828 (0.818) | 1.247 (0.734) | |
| n: number of samples; bp^a^: no. of usable basepairs (loci <5.0% missing data); π: nucleotide diversity (average over locus); S: No. of sites with substitutions; *h*: gene diversity; SSD: Sum of Squared Deviations; HRI: Harpending's Raggedness Index. For Tajima’s D, Fu’s Fs and expansion models a *P*<0.05 is significant (*). ^1^ = The significant Tajima’s D of clade I-VII of the concatenated cpDNA dataset had a *P-*value of 0.0499. | | | | | | | | | | | | |  |


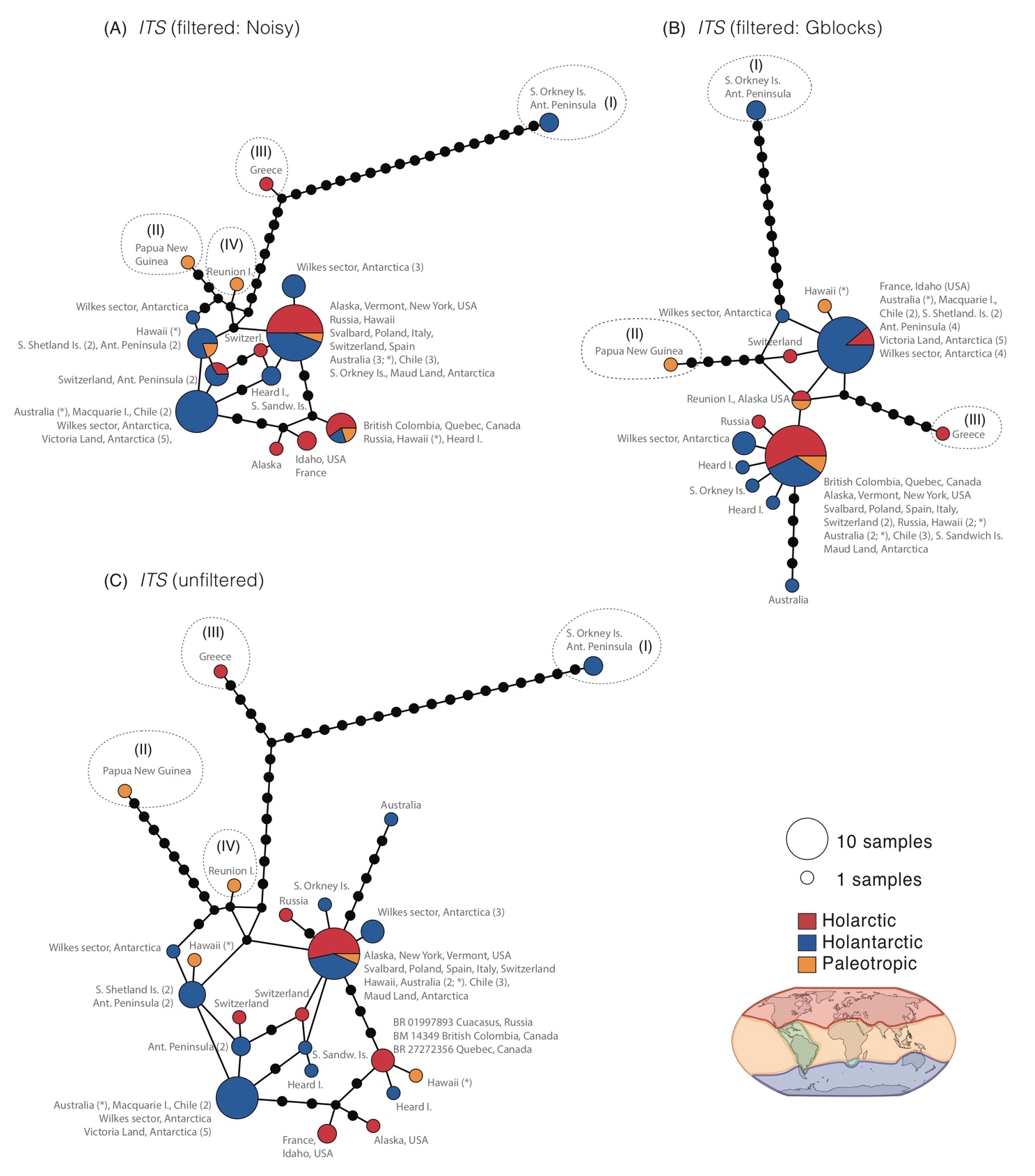


**Supplementary Fig. S3.** Haplotype networks of *ITS* within the species *Ceratodon purpureus,* after treatment with (A) NOISY, (B) Gblocks or as (C) original data. Haplotype circle sizes and colours correspond to the number of specimens and globally recognised bryofloristic kingdoms (see legend; Schofield, 1992), respectively. Branches represent mutations between haplotypes, with mutations shown as 1-step edges. Multiple copies of *ITS* derived from the same specimens (in samples from Hawaii and Australia) are indicated with (*).

**Supplementary Table S3.** Fst (below diagonal) and Φst (above diagonal) of *Ceratodon purpureus* samples of latitudinal and longitudinal geographically divided areas, including samples from the ‘concatenated cpDNA’ dataset. The number of samples (n) per geographic area is given in the top row. P values are represented by * for P<0.05 and ** for P<0.01.

| Latitudinal | Φst  Fst | >30°N (n=18) | 30°S - 30°N (n=9) | >30°S (n=20) |
| --- | --- | --- | --- | --- |
|  | >30°N | - | 0.197 (0.015*) | 0.153 (0.002**) |
|  | 30°S - 30°N | 0.195 (0.001**) | - | 0.186 (0.006**) |
|  | >30°S | 0.244 (0.000**) | 0.176 (0.001**) | - |
| Longitudinal | Φst  Fst | 30°W-165°W (Americas) (n=27) | 165°W-60°E (Australia/Asia) (n=9) | 60°E-30°W (Africa/Europe) (n=11) |
|  | 30°W-165°W | - | 0.084 (0.070) | 0.036 (0.153) |
|  | 165°W-60°E | 0.149 (0.001**) | - | 0.013 (0.186) |
|  | 60°E-30°W | 0.144 (0.000**) | 0.133 (0.002**) | - |

**Supplementary Table S4.** Fst (below diagonal) and Φst (above diagonal) of *Ceratodon purpureus* samples of latitudinal and longitudinal geographically divided areas, including samples from the GBLOCKS filtered *ITS* dataset. The number of samples (n) per geographic area is given in the top row. P values are represented by * for P<0.05 and ** for P<0.01.

| Latitudinal | Φst  Fst | >30°N (n=17) | 30°S - 30°N (n=6) | >30°S (n=33) |
| --- | --- | --- | --- | --- |
|  | >30°N | - | 0.003 (0.398) | 0.087 (0.010**) |
|  | 30°S - 30°N | 0.184 (0.006**) | - | 0.016 (0.269) |
|  | >30°S | 0.160 (0.000**) | 0.069 (0.022*) | - |
| Longitudinal | Φst  Fst | 30°W-165°W (Americas) (n=24) | 165°W-60°E (Australia/Asia) (n=19) | 60°E-30°W (Africa/Europe) (n=13) |
|  | 30°W-165°W | - | -0.004 (0.516) | 0.017 (0.245) |
|  | 165°W-60°E | 0.064 (0.000**) | - | 0.051 (0.081) |
|  | 60°E-30°W | 0.150 (0.000**) | 0.170 (0.000**) | - |

**Supplementary Table S5.** Analysis of Molecular Variance (AMOVA) within *Ceratodon purpureus* populations based on pairwise difference among and within populations at the haplotypic (Fst) and nucleotidic level (Φst), as calculated in the ‘concatenated cpDNA’ dataset (upper) and GBLOCKS filtered *ITS* dataset (lower). Populations are based on latitudinal and longitudinally separated geographic areas (see manuscript and Table S4.1 and S4.2 for latitudinal and longitudinally divided regions, respectively, as well as the number of individuals per area). P values are represented by * for P<0.05 and ** for P<0.01.

|  |  | Latitudinal | | Longitudinal | |
| --- | --- | --- | --- | --- | --- |
|  |  | Fst | Φst | Fst | Φst |
| cpDNA | Among populations | 21.44 | 17.13 | 14.39 | 5.67 |
| (average over 53 loci) | Within populations | 78.56 | 82.87 | 85.61 | 94.33 |
|  | P-value | 0.000** | 0.000** | 0.000** | 0.066 |
| ITS (GBLOCKS) | Among populations | 14.17 | 6.21 | 11.73 | 1.47 |
| (average over 80 loci) | Within populations | 85.83 | 93.79 | 88.27 | 98.53 |
|  | P-value | 0.000** | 0.034* | 0.000** | 0.222 |


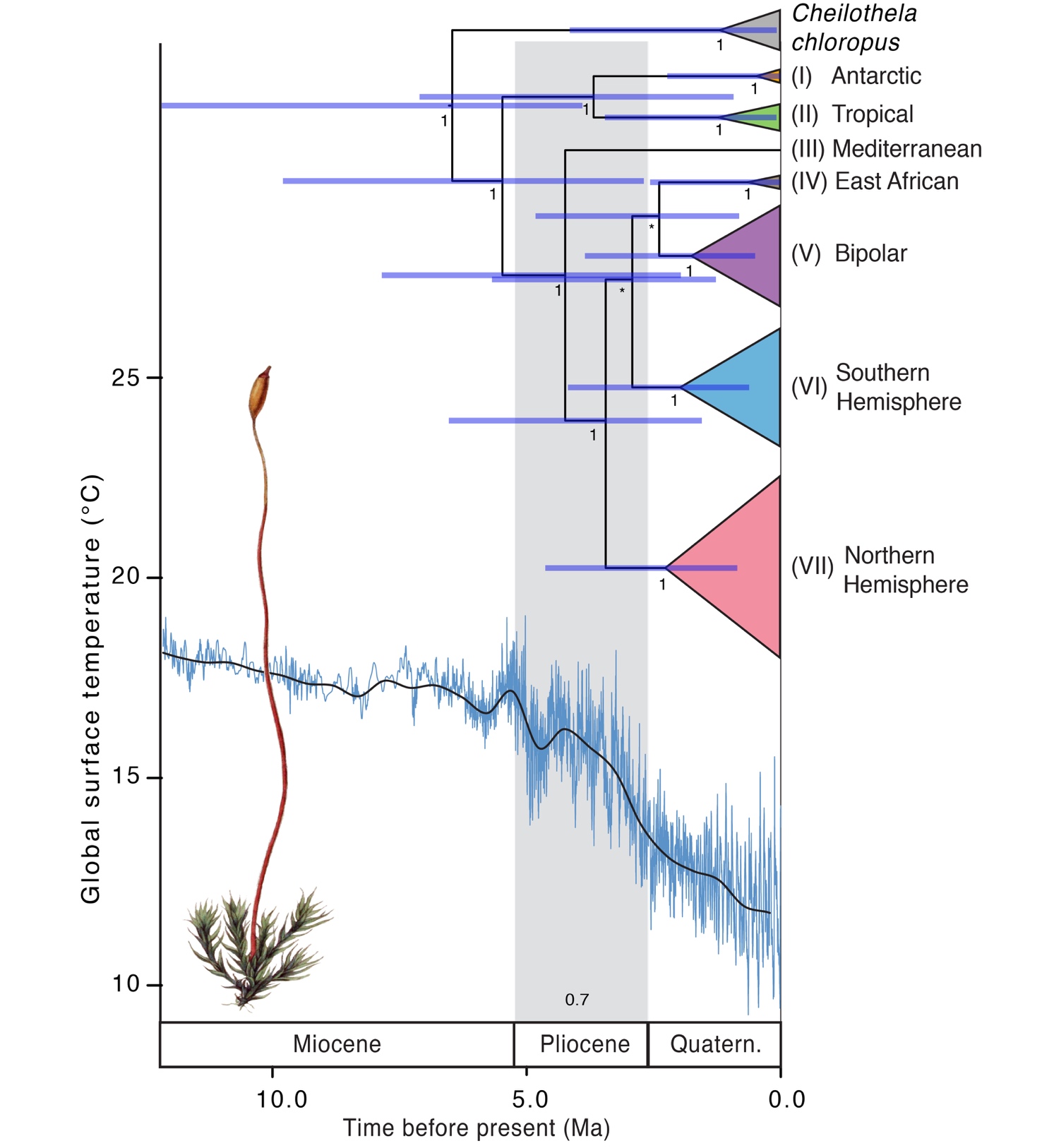


**Supplementary Fig. S4.** Time-calibrated phylogeny of *Ceratodon purpureus* using a Yule tree prior. All other analysis settings and tree representations are the same as in Fig. 5, showing the BEAST analysis using a coalescent tree prior.
